# A model of risk and mental state shifts during social interaction

**DOI:** 10.1371/journal.pcbi.1005935

**Published:** 2018-02-15

**Authors:** Andreas Hula, Iris Vilares, Terry Lohrenz, Peter Dayan, P. Read Montague

**Affiliations:** 1 Austrian Institute of Technology, Vienna, Austria; 2 Wellcome Trust Centre for Neuroimaging, University College London, London, United Kingdom; 3 Human Neuroimaging Laboratory, Virginia Tech Carilion Research Institute, Roanoke, Virginia, United States of America; 4 Gatsby Computational Unit, University College London, London, United Kingdom; 5 Department of Physics, Virginia Polytechnic Institute and State University, Blacksburg, Virginia, United States of America; Harvard University, UNITED STATES

## Abstract

Cooperation and competition between human players in repeated microeconomic games offer a window onto social phenomena such as the establishment, breakdown and repair of trust. However, although a suitable starting point for the quantitative analysis of such games exists, namely the Interactive Partially Observable Markov Decision Process (I-POMDP), computational considerations and structural limitations have limited its application, and left unmodelled critical features of behavior in a canonical trust task. Here, we provide the first analysis of two central phenomena: a form of social risk-aversion exhibited by the player who is in control of the interaction in the game; and irritation or anger, potentially exhibited by both players. Irritation arises when partners apparently defect, and it potentially causes a precipitate breakdown in cooperation. Failing to model one’s partner’s propensity for it leads to substantial economic inefficiency. We illustrate these behaviours using evidence drawn from the play of large cohorts of healthy volunteers and patients. We show that for both cohorts, a particular subtype of player is largely responsible for the breakdown of trust, a finding which sheds new light on borderline personality disorder.

## Author summary

In multi-round games in which players can benefit by trusting each other, swift and catastrophic breakdowns can arise amidst otherwise efficient cooperation. We present a model that quantifies this as a form of anger, and we exploit novel algorithmic improvements in inference based on the model to examine exchanges involving healthy volunteers and people suffering from personality disorders. This provides a new view on the problems that can underlie social interactions.

## Introduction

Assessing the internal characteristics of another person is a fundamental requirement for success in human social decision making. Neither people’s self-reports, nor any current measurement device provides complete, veridical, information about another person’s state. Nevertheless, we are typically quite adept at inferring the preferences and intentions of others and even at manipulating their states, in both cases over the course of multi-round interactions. One way to formalize this capacity is via the so-called interactive Partially Observable Markov Decision Process (I-POMDP; [1]). This is a regular Markov Decision Process (see [2]) augmented with (a) partial observability (see [3]) about the characteristics of a partner; and (b) a notion of cognitive hierarchy (see [4, 5]), associated with the game theoretic interaction between players who model each other.

In recent work, we used approximate inference methods in the I-POMDP to capture the effect of an other-regarding utility preference (namely guilt) in modeling behaviour in a popular multi-round trust task (MRT) [6–10]. This model offered powerful accounts of both the behavior of subjects, and also aspects of their neural activity [8, 9].

However, a detailed inspection of the residual errors revealed two key characteristics that were missing from the model: social risk aversion and irritation. Here we formalize both, including extending the I-POMDP framework to encompass the possibility that players might change their internal states as a result of interactions. We thereby fit subjects’ choices much more closely.

First, investors are dominant in the MRT, in that they can still make substantial sums of money based on initial endowments in each round without investing anything. Perhaps as a result of this, we observed that some investors apparently treat a portion of their endowment as being exclusively theirs; only risking the remainder in the social exchange. This is a form of social preference that is absent in the Fehr-Schmidt model of other-regarding preferences that we adopted as our baseline model [11]. Here, we treat it explicitly as a form of (social) risk aversion, a factor that has previously been considered in terms of this task [12].

A second, and more complicated, failure of the existing model is that sample investment profiles are generally too homogeneous. That is, as pointed out in some of the early neuroeconomics studies of the MRT [6], cooperation between the players can readily break down in the face of apparent defection; with coaxing then being necessary to reestablish it (especially on the part of trustees). Such phenomena appear particularly prevalent in play involving subjects suffering from psychiatric conditions such as borderline personality disorder (BPD) (see for instance [6]). This condition is frequently characterised by difficulties in maintaining social relationships, sudden ruptures in trust, and social withdrawal or aggression (see [13, 14]).

We therefore augmented the model with a form of irritation. When irritated, subjects can exhibit substantially different rules of behaviour, for instance being unwilling to cooperate at all, and reducing their depth of interpersonal reasoning. This leads to breakdowns in cooperation. To predict what might happen in response to their own choices, and thus, if beneficial to them, to prevent a breakdown, subjects need to model the possibility of such a shift in their partner’s state. They can then change their behavior prospectively.

As originally defined however, the I-POMDP framework explicitly excluded the possibility of one agent’s actions changing the reward evaluation function of the other agents directly (see p.57–58, [1]). This non-manipulability assumption is also in keeping with the conventional Bayes-Nash model [15], in which nature allocates an agent’s preference type before interactions start, and other agents merely make inferences about that type based on their observations. We extended the I-POMDP framework to encompass the possibility of internal state shift manipulations, and indeed that other agents may be aware or unaware of the possibility of such shifts or the exact actions that might trigger them. This then gives rise to much richer dynamics and more intricate manipulations during social exchange.

We generated simulated data using our extended model to show how the inclusion of these dimensions of social manipulation affects the course and understanding of human social exchange, and to validate parameter identifiability. We then demonstrated how the new mechanisms allow us to account for behaviour that appeared anomalous according to our previous model. Finally, we identify parameter settings associated with breakdowns and show that this characteristic is enriched in the population of BPD subjects [6]. This provides new insights into the workings of such disorders.

### Background

#### Trust task

**Fig 1:**
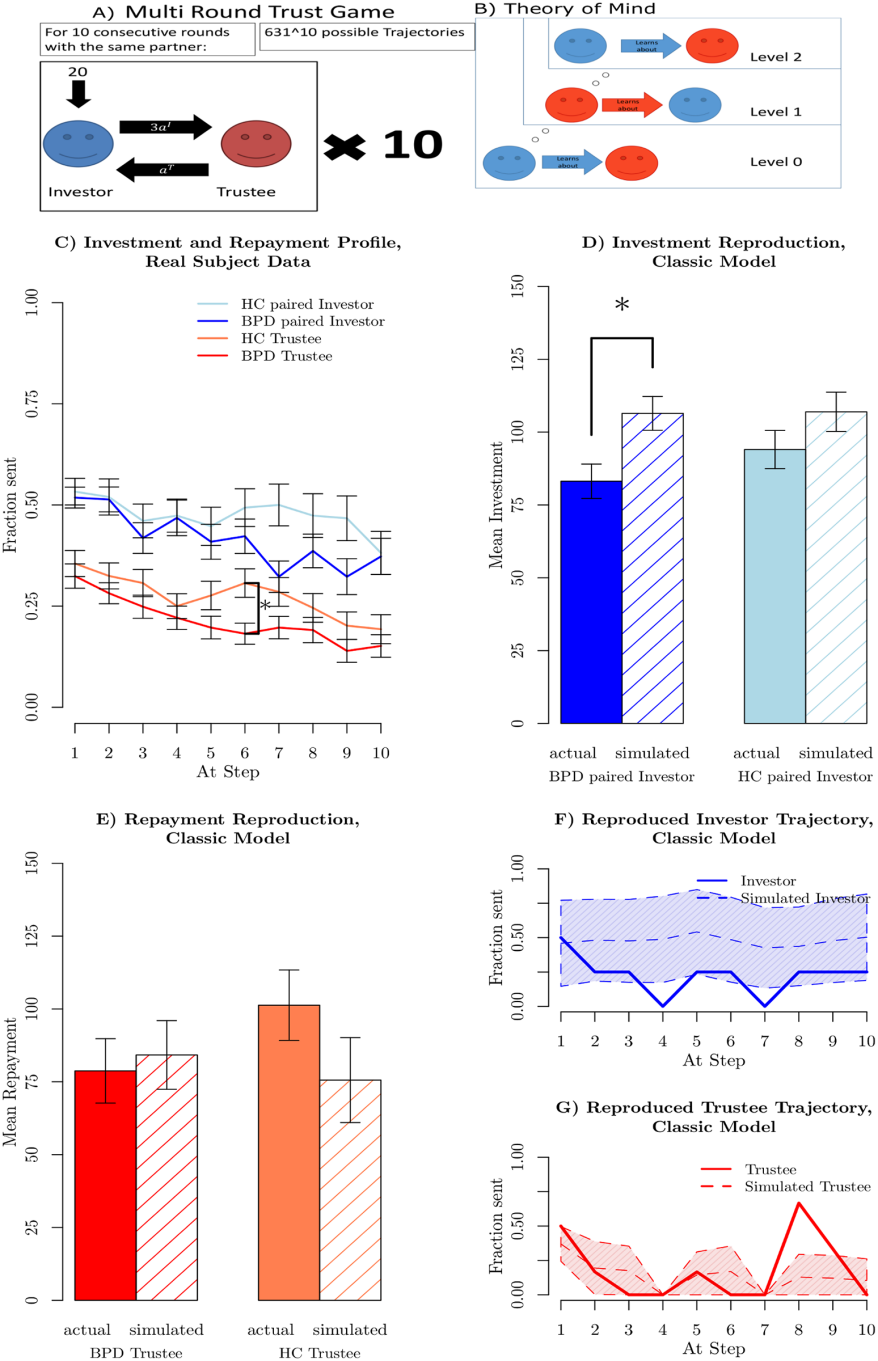
Basic game and game data features. A: Physical features of the multi round trust game. B: Recursive reasoning about a partner. At level 0 the blue player learns about the partner. At level 1 the blue players knows that the red player learns about them too (that is, that the red player is level 0). At level 2 the blue player knows that the red player knows they are learning about them (i.e. that the red player is level 1 and thinks of the blue player as level 0). This recurses up to higher levels. C) Averaged investments and repayments in the data set. Errorbars show standard errors of the mean. An asterisk denotes the largest difference (*p* = 0.05, two sided permutation t-test) corrected for multiple comparisons at the 10 steps. D) Average investment in real and in simulated exchanges based on best fit parameters. An asterisk denotes a significant difference (*p* < 0.05, two sided permutation t-test) in means between the original data and the generated exchanges. E) Average repayments in real and in simulated exchanges based on the actual parameters. F) Sample trajectory for an investor vs average of 200 generated exchanges with best fitting parameters, based on the earlier model (see [10]). Shaded area shows estimated standard deviations. G) Sample trajectory for a trustee vs average of 200 generated exchanges with best fitting parameters, based on the earlier model (see [10]). Shaded area shows estimated standard deviations.

The multi round trust task (see [9, 10, 16]), based on work by McCabe et al. (see [17]) (see Fig 1A) is a paradigmatic social exchange game. We redescribe it here:

It involves two people, one playing the role of an “investor” the other that of a “trustee”, over 10 sequential rounds. Quantities pertaining to the investor and trustee are denoted by superscripts “I” and “T” respectively. The participants played at the same time but did not know or meet each other at any point.

Both players know all the rules of the game. In each round, the investor receives an initial endowment of 20 monetary units. The investor can send any *a*^*I*^ units of this amount to the trustee. The experimenter triples this quantity and then the trustee decides how much (an amount *a*^*T*^) to send back to the investor. This amount must be between 0 points and the whole amount that she receives. The repayment by the trustee is not increased by the experimenter. After the trustee’s action, the investor is informed, and the next round starts. On each round the financial payoffs of the two actors can be calculated: for the investor this is:

$$\begin{array}{cc} \chi^{I}\left(a^{I},a^{T}\right) & =20-a^{I}+a^{T} \end{array}$$ (1)

and for the trustee:

$$\begin{array}{cc} \chi^{T}\left(a^{I},a^{T}\right) & =3a^{I}-a^{T}. \end{array}$$ (2)

For computational simplicity, the model treated the possible choices on a coarser grid, allowing for five investor actions and five corresponding trustee reactions. The five investor actions correspond to investing 0, 5, 10, 15 or 20 or $\left\{0,\frac{1}{4},\frac{1}{2},\frac{3}{4},1\right\}$ of their endowment, while the trustee responses correspond to the return of 0, $\frac{1}{6}$, $\frac{1}{3}$, $\frac{1}{2}$ or $\frac{2}{3}$ of the received amount. The case in which the investor gives 0 is special, since the trustee has no choice but to return 0. We round real subject actions to the respective nearest grid point.

The Nash equilibrium (see [18], here based on pure monetary outcomes) for this game mandates a trivial interaction. That is because, in the last round, the investor should never invest anything, since the trustee could defect without punishment. Thus the interaction progressively unravels. Real subject behaviour in the game is quite different, and typically leads to substantial investments and returns.

#### Generative model

A generative model of the multi round trust task was introduced in earlier work (see [10]); we enrich it here. Parameters that agents are assumed to learn about (via Bayesian inference) over the course of the interaction, are called “intentional”; the other parameters are inferred by the experimenter through the process of fitting the choices (using maximum likelihood), but are merely assumed by the subjects and are constant throughout the experiment.

The foundation of subjects’ payoff evaluation was modeled by the Fehr-Schmidt inequality aversion utility ([11]):

The intentional parameters *α*^*I*^, *α*^*T*^ ∈ {0, 0.4, 1} quantify guilt (see [11]), and change subjects’ utility functions from those described above for the investor to

$$\begin{array}{c} r^{I}\left(a^{I},a^{T},\alpha^{I}\right)=\chi^{I}\left(a^{I},a^{T}\right)-\alpha^{I}\max\left\{\chi^{I}\left(a^{I},a^{T}\right)-\chi^{T}\left(a^{I},a^{T}\right),0\right\}, \end{array}$$

and for the trustee to

$$\begin{array}{c} r^{T}\left(a^{I},a^{T},\alpha^{T}\right)=\chi^{T}\left(a^{I},a^{T}\right)-\alpha^{T}\max\left\{\chi^{T}\left(a^{I},a^{T}\right)-\chi^{I}\left(a^{I},a^{T}\right),0\right\}. \end{array}$$

High guilt *α* = 1 means that every point of advantageous inequality in payoffs diminishes the utility of the outcome by 1 i.e. there is no felt benefit from having a larger payoff than the partner. A low guilt of 0 means that only raw outcome maximization is relevant to the agent, while 0.4 is a more measured, but mostly self-interested agent.

The proximal cause of behaviour is a set of reward expectations *Q*(*a*, *h*) for taking a given action *a* after having experienced a history of events *h* in the game. Here the agent was supposed to learn about the other agent from this history *h*, following Bayes rule from a given initial belief system. These are assumed to generate choices using a softmax rule (something that is known to all parties) (see examples in [19–22])

$$\begin{array}{c} \pi \left(a,h\right)=P\left[a|h\right]=\frac{e^{\beta Q(a,h)}}{\sum_{b\in A}e^{\beta Q(b,h)}} \end{array}$$ (3)

where *β* > 0 is called the inverse temperature parameter and controls how diffuse are the probabilities. The policy

$$\begin{array}{c} \pi \left(a,h\right)=\{\begin{array}{cc} 1 & \text{if}\;Q(a,h)=\max\left\{Q(b,h)|b\in A\right\}\;(\text{assuming}\;\text{this}\;\text{is}\;\text{unique})\\ 0 & \text{otherwise} \end{array} \end{array}$$ (4)

can be obtained as a limiting case for *β* → ∞.

Subjects were assumed to use Bayesian inference to infer their partners’ guilt over the course of the interaction. This is possible since a high guilt (*α* = 1) partner will provide high investments or returns and appear persistently cooperative, while a low guilt (*α* = 0) partner is likely just to maximise their own winnings (and so only cooperate for Machiavellian reasons). The intermediate setting (*α* = 0.4) of being “mostly selfinterested” exists to provide somewhat less extreme exploitation patterns (see [10] for details), while values of *α* ≥ 0.5 produce essentially the same patterns, generatively, as *α* = 1. Hence, we only use the above mentioned 3 levels.

Agents know their own guilt; but adopt a multinomial distribution on the possible guilt values of their partner, with a Dirichlet prior on probabilities of the multinomial distribution. Thus the initial belief state ($B_{0}$) is a symmetric Dirichlet-Multinomial distribution,

$$\begin{array}{c} B_{0}∼DirMult\left(d_{0}\right),\;d_{0}=\left(1,1,1\right). \end{array}$$

To be consistent with preceding work (see [9]), the posterior distribution is approximated as a Dirichlet-Multinomial distribution with the parameters of the Dirichlet prior being updated to

$$\begin{array}{c} d_{t+1}^{i}=d_{t}^{i}+P\left[o_{t+1}=\text{observed}\;\text{action}|\alpha^{\text{partner}}=\alpha_{i}\right]. \end{array}$$

Next, a player could be aware that their partner was also learning about them, a recursive concept formalized as computational theory of mind (ToM) or reasoning level *k*, and depicted in Fig 1B. A level 0 investor learns about the trustee, but treats her as being random rather than intentional. A level 1 investor treats the trustee as being level 0, implying that the trustee is assumed to learn about a non-intentional investor. A level 2 investor treats the trustee as being level 1, implying that the trustee is assumed to know that the investor is learning about them too. This continues recursively. One consequence of the interplay of I-POMDP modeling and the asymmetric nature of the game is that only even levels yield new insight into investor behaviour, and only odd levels into that of the trustee (see [10]). In the original model, computational considerations restricted the theory of mind to *k*^*I*^ ∈ {0, 2} for the investor and *k*^*T*^ ∈ {0, 1} for the trustee. In the MRT, levels of ToM higher than 4 of ToM do not appear to yield qualitatively new behavioural patterns (see supplementary material S1 Text “Theory of Mind Limitation”), and so we extended consideration to levels {0, 2, 4} for investors and {0, 1, 3} for trustees.

At the lower end of the ToM ladder, the level *k* = −1 agent (i.e. the partner model of a level 0 agent) assumes equal probability for all partner guilt states and neither learns nor plans for the partner’s decisions, only using immediate expected utilities. However, the more complex agents learn and make recursive inferences about their partners.

Finally, subjects were classified according to their planning capacity *P*, which quantifies how many steps of the future of the interaction they take into account when assessing the consequences of their actions. In the original model, this could take the values *P* ∈ {0, 2, 7}. However, it turns out that play for *P* = 4 has very similar features to that of *P* = 7, involving exploitation of the partner and inhomogeneous effects caused by the horizon of the game (see supplementary material S1 Text “Planning”). Therefore, to liberate the computational capacity to model an additional intentional parameter, we restricted *P* to {1, 2, 3, 4}.

The inverse temperature parameter of the softmax that was fixed at $\beta =\frac{1}{3}$ in the original model, was here fit using values $\beta \in \{\frac{1}{4},\frac{1}{3},\frac{1}{2},1\}$. Note the relatively large numerical values of investment and return, which is why the inverse temperatures may seem relatively small compared with other studies.

To gauge the differences between models with different numbers of parameters, we used Likelihood ratio tests, since all appearing models are ultimately nested. Our requirement for a meaningful change was that in a likelihood ratio test the probability of the data being generated by a simpler nested model should be below *p* < 0.05, compared to the more complex model. The test is defined using the negative loglikelihood (NLL) $-\text{log}\;\mathbb{P}[x_{s}|\theta_{s}^{*};M]$ at the best fitting parameters $\theta_{s}^{*}$ for each subject *s* under the given model *M* and comparing this to the NLL under a richer model *M*_+_ and its best fitting parameters $\vartheta_{s}^{*}$ against a *χ*^2^ statistic:

$$\begin{array}{c} \sum_{\text{subjects}\;s}2(\log\;P\left[x_{s}|\theta_{s}^{*};M\right]-\log\;P\left[x_{s}|\vartheta_{s}^{*};M_{+}\right])>\chi_{1-p,f}^{2}. \end{array}$$ (5)

Here *f* denotes the difference in degrees of freedom between the 2 models. This criterion decided in favour of all appearing models, see the supplementary material S1 Text “Model Selection” for details.

We agumented these considerations by the Bayesian Information criterion (BIC, see [23]), which penalizes the number of parameters *n* used to fit each subject according to the number *m* of data points obtained in each exchange.

$$\begin{array}{c} \text{BIC}\left(M\right)=\sum_{\text{subjects}\;s}(-2\log\;P\left[x_{s}|\theta_{s}^{*};M\right]+n(\log\left(m\right)-\log\left(2\pi \right)) \end{array}$$ (6)

In the multi round trustgame *m* = 10, due to the 10 choices per subject. The correction factor is for small *m* [24]. A BIC based comparison of all models in this work can be found in the supplementary material (S1 Text “Model Selection”).

The parameters of the final model can be seen in Table 1.

**Table 1:** Table of model parameters, ranges and parameter meaning.

Parameter	Values	Concept
Guilt *α*	{0, 0.4, 1}	Measure of tendency to try and reach a fair outcome.
Plan *P*	{1, 2, 3, 4}	Number of steps likely planned ahead.
Theory of Mind *k*	{0, 2, 4} or {0, 1, 3}	number mentalisation steps.
Inverse Temperature *β*	$\left\{1,\frac{1}{2},\frac{1}{3},\frac{1}{4}\right\}$	Certainty of own choice preference.
Risk Aversion (Belief) *ω* (*b*(*ω*))	{0.4, 0.6, 0.8, 1.0, 1.2, 1.4, 1.6, 1.8}	Value of money kept over (potential) money gained.
Irritability *ζ*	{0, 0.25, 0.5, 0.75, 1.0}	Tendency to retaliate on worse than expected partner actions.
Irritation Belief *q*(*ζ*)	{0, 1, 2, 3, 4}	Initial belief on likelihood of the partner being irritable.

Short description of all Parameters in the full model.

### Subject data

We use the data set shown in King-Casas et al. (see [6]), consisting of 93 healthy investors, paired with 93 trustees, of which 55 were BPD diagnosed trustees (BPD Group, “BPD”) and 38 were healthy trustees, matched in age, gender, IQ and socio-economic status (SES) with the BPD trustee group (healthy control group, “HC”). The precise demographics can be found in King-Casas et al.

## Results

We start by illustrating the failures of the existing model of the task. These motivate the changes that we then describe.

### Model failure

Fig 1C shows the average investments and returns in the data from King-Casas et al. [6]. The dark blue and dark red lines in Fig 1C show the respective average investments and returns for healthy investors playing BPD trustees. The lighter blue and red lines show average investments and returns for healthy investors and healthy trustees who matched the BPD trustees in socio-economic status (SES), IQ, age and gender. Investments averaged about half the initial endowment and evolved over trials. In the second half of the game, investors paired with BPDs invested considerably less than investors paired with healthy trustees. This effect was a central topic in King-Casas et al, and was explained by BPD trustees not heeding warning signals from their investor partners, indicating investor dissatisfaction with the BPD patients’ lack of reciprocation. The strongest difference (*p* = 0.05, two sided permutation t-test, Bonferroni corrected for 10 time step comparisons, indicated by an asterisk in Fig 1C in trustee reciprocation at step 6 also indicates the time at which the average investment trajectories have persistently diverged. This gave rise to the difference in early vs late investment between the two groups that was reported in King-Casas et al. [6].

The solid bars in Fig 1D show the average total investments in the real data for the two groups. The hatched bars show the result of generating data from the model in our earlier work (see [10]) (using the extensions discussed above to higher theory of mind and lower maximal planning). Model data is generated for each dyad, using that dyad’s best fitting parameters. The model overestimates the investments of the BPD-paired investors by about 30%.

Fig 1E demonstrates a similar issue for the modelled trustees. The simulated HC trustees (hatched bars) return less than the actual HC trustees. Although it may seem that the simulated BPD trustees return similar proportions to the actual BPD trustees, this actually flatters the model, since this repayment is based on the over-generous model investment (the hatched bars in part D) rather than the true, more miserly, investment.

A second model failure concerns the detailed dynamics of investment across the task. The solid lines in Fig 1F and 1G show a selected sample interaction between a healthy investor (see Fig 1F) and a BPD trustee (see Fig 1G). The trustee provides a poor return in trial 3, and is met by zero investment in trial 4. The same pattern repeats in trials 6 and 7. The trustee is then far more generous in trial 8; this then coaxes (to adopt a term from a former study, see [6]) the investor to continue investing, though after 2 breaks, the investors is unwilling to much increase their investment above a low level. The trustee then defects on trial 10, returning nothing. The conclusion was that a significant portion of the BPD group lacked mechanisms that could consistently repair the faltering interactions that occur when subjects become what we will describe as being irritated. Thus tentative ruptures (in the form of drops in investment level) turned into complete breaks, with the investor using their position of power in the game to punish the trustee.

The dashed lines in Fig 1F and 1G show the result of simulating 200 trajectories using parameters fit to the actual data, and also making predictions at each step based on the actual investments and returns of the dyad prior to each step (explaining why the model return is also 0 on trials 4 and 7). The shaded areas show the empirical standard deviations—which are evidently very wide. In fact, the specific reductions are not only absent in the averages; the modelled investment following the trustee’s defection on trials 3 and 6 decreased to 0 on only 11% and 13.5% of the sample runs; compared with the collapse to 0 apparent in the actual data.

We addressed these sources of model failure by introducing two new parameters, associated with risk aversion and irritation.

### Risk aversion

The investor is in charge in the MRT, since she could simply keep her endowment on each round. It has been noted since the advent of this kind of trust game (see [17]) that a lack of investment could represent a social form of risk aversion rather than a lack of trust (see [12]). This could account for differences in levels of investment regardless of the cooperativity of either partner.

We parameterize such risk aversion as a multiplicative factor *ω*^*I*^ in the payoff functions, increasing or decreasing the evaluation of money that the investor keeps for herself compared to the money returned by the trustee:

$$\begin{array}{c} \chi_{\omega }^{I}\left(a^{I},a^{T}\right)=\omega^{I}\left(20-a^{I}\right)+a^{T}, \end{array}$$ (7)

with *ω*^*I*^ ∈ [0.4, 1.8] (in 7 steps of 0.2). The trustee is subordinate in the task, and so does not have a risk parameter of their own. Instead, the trustee makes an assumption about the investor’s degree of risk aversion, at one of the above mentioned 8 values. We capture intentional aspects of trust through guilt, and so treat risk aversion as a non-intentional parameter. However, in keeping with Harsanyi [15], both players are assumed to be consistent, with the investor believing the trustee to know her risk aversion, and to know that she believes this; and the trustee believing that the investor believes this too. We write *b*^*T*^(*ω*^*I*^) for the trustee’s belief about the investor’s value of *ω*^*I*^.

Depending on the trustee’s belief *b*^*T*^(*ω*^*I*^), there will be either earlier or later attempts at exploitation. If *b*^*T*^(*ω*^*I*^) < 1, then the trustee infers the investor will keep investing, even if the trustee has been relatively uncooperative (i.e. the investor will be risk-seeking). Conversely, if *b*^*T*^(*ω*^*I*^) > 1, then the trustee will infer that any investment is contingent on their behavior, and there could be negative consequences of poor return. For values *b*^*T*^(*ω*^*I*^) > 1.4, the trustee expects the investor to invest so little that building up trust will not be worthwhile in the first place. In this case, the interaction will rupture.

Illustrations can be found in the supplemental material S1 Text “Risk Aversion”, along with additional detail on the workings of this parameter.

Including risk aversion allows the model to account for the behavioural data much more proficiently, with the average Investor NLL improving from 12.96 to 9.68. The average trustee NLL improves from 11.37 to 9.5. In terms of likelihood ratio tests the model with risk aversion is a better model for the observed data at a threshold of *p* < 10^−46^ on the investor side and *p* < 10^−11^ on the trustee side. The average BIC for the investors improves from 27.3 to 21.68, and for the trustees, from 24.1 to 21.4. A BIC based comparison of all models in this work can be found in the supplementary material (S1 Text “Model Selection”).

### Irritation

We explained the breakdown in cooperation evident in Fig 1F and 1G as arising when the participants become irritated. Formalizing this leads to four considerations: (i) what do subjects do differently when irritated; (ii) what leads a subject to become irritated; (iii) how can irritation be repaired; (iv) and what do subjects know about their own irritability? We offer a highly simplified characterization of all four of these. Individual interactions in the 10 round MRT are too short to license more complex treatments.

**Definition 1 (Irritability)** *We define the irritated state as associated with planning* *P* = 0, *guilt* *α* = 0, *temperature* $\beta =\frac{1}{2}$ *and complete disregard of beliefs about the other player that have hitherto been established. Additionally, for investors, the value of the risk aversion under irritation* ($\omega_{\iota }^{I}$) *is bounded below at* 1.0 *i.e.* $\omega_{\iota }^{I}=\text{max}\{1.0,\omega^{I}\}$, *since otherwise “irritated” investors may not show punishing behaviour. We model the players’ policy* *π as being a mixture between irritated* *π*_*ι*_ *and the nonirritated* $\pi_{\tilde{\iota }}$ *choices, with irritation weight* *v*_*ι*_

$$\begin{array}{c} \pi \left(a,h\right)=\left(1-v_{\iota }\right)\pi_{\tilde{\iota }}\left(a,h\right)+v_{\iota }\pi_{\iota }\left(a,h\right). \end{array}$$

*A participant’s irritation weight is assumed to start at* *v*_*ι*_ = 0, *and to increase when their partner’s action (investment or return) falls short of the value expected on the basis of the current model they have of the partner (including the partner’s potential irritation):*

$$\begin{array}{c} v_{\iota }=\min\{v_{\iota }+\zeta ,1.0\}\;given\;unfavorable\;investment\;or\;return \end{array}$$ (8)

*where* *ζ* *is a subject-specific parameter. Irritation decreases through a process of repair when the action exceeds this expected value*

$$\begin{array}{c} v_{\iota }=\max\{v_{\iota }-\zeta ,0.0\}\;given\;favorable\;investment\;or\;return \end{array}$$ (9)

**Definition 2 (Intentional Inference about Irritation)** *Players maintain and constantly update beliefs about the partner’s irritability during the interaction in exactly the same way as about the partner’s guilt: that is, they employ a Dirichlet prior on a multinomial distribution over five possible irritation values* *ζ* ∈ {0, 0.25, 0.5, 0.75, 1} *(dubbed respectively “nonirritable” and four different“irritable” types in the following) and use the same approximate inference rule as is used for guilt. In particular, this means that they represent the possible current values of* $v_{\iota }^{\text{partner}}$, *that is, the partner’s current degree of irritation at the given choice, marginalizing over the posterior distribution over* *ζ*.

However, unlike guilt, for which we consider only one (uniform) initial belief setting, we consider a discrete set of possible prior beliefs about irritability. That is, irritability awareness is treated as an additional discrete new parameter (*q*^*I*^(*ζ*^*T*^); *q*^*T*^(*ζ*^*I*^) ∈ {0, 1, 2, 3, 4}). The investor’s value *q*^*I*^(*ζ*^*T*^) determines prior weights of his belief over the trustee’s actual irritability *ζ*^*T*^. The trustee’s value *q*^*T*^(*ζ*^*I*^) determines prior weights of her belief over the investor’s actual irritability *ζ*^*I*^. These priors are intended to cover a suitable range of possibilities; as noted, the MRT involves too few choices to license a richer depiction.

Table 2 lists the four particular prior beliefs *q*(*ζ*) over the values of irritation. Players range from being *ignorant* about the possibility that their partners might be irritable, through stages of *optimism* that they are not, *realism* that they could be, *pessimism* that they likely are and *fatalistic* that they certainly are.

**Table 2:** Table of irritation prior parameters, based on the awareness parameter *q*(*ζ*).

belief	prior over *ζ*	descriptor
*q*(*ζ*)	{0.0, 0.25, 0.5, 0.75, 1.0}	
0	{400.0, 0.1, 0.1, 0.1, 0.1}	ignorant
1	{4.0, 0.5, 0.5, 0.5, 0.5}	optimistic
2	{0.4, 0.1, 0.1, 0.1, 0.1}	realistic
3	{2.0, 1.0, 1.0, 1.0, 1.0}	pessimistic
4	{0.1, 0.1, 0.1, 0.1, 400.0}	fatalistic

Irritability belief settings.

Finally, although we assume that players infer both their partner’s inequality aversion and their partner’s irritability level during the interaction, we do not allow subjects to consider their *own* future irritation. This follows famous (see [25]) observations of subjects’ inability whilst engaging in ‘cold’ cognition to contemplate the possibility of one’s own behaviour under ‘hot’ cognition (i.e. in the affective state). “Cold” cognotion describes a emotional state, in which the subject is not under influence of particular strong emotions or cravings (hunger, thirst, fear, anger for example), while “hot” states are a model of a subject acting under the influence of such factors.

In the case of our model, all agents start in the “cold” (nonirritated) state, yet irritable agents can transition into the “hot” state of decision making under irritation. An irritable, but not currently irritated, agent is not modeled to consider their prospective actions under irritation in our case, indeed our approach makes them unaware of their own irritability. This is an direct example of the “prospective” variety of the “cold-to-hot” empathy gap mentioned on p49. of the cited work [25].

**Fig 2:**
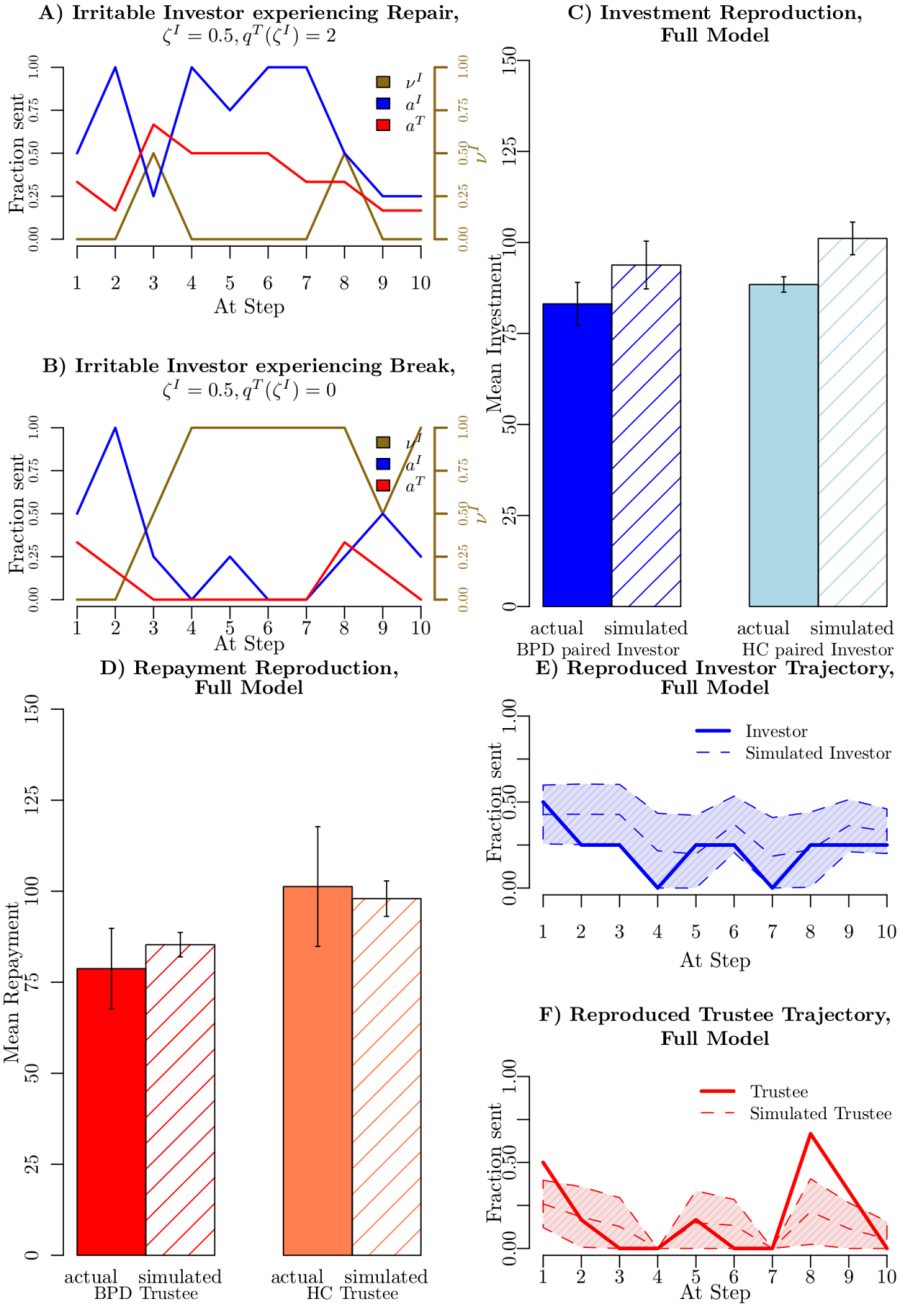
Irritation mechanism and resulting data reproduction. A) Simulated Repair Interaction. Single trajectory of two aware players (blue for investor, red for trustee). The golden line depicts the evolution of the investor irritation weight during the interaction. B) Simulated Break Interaction. Both players were irritability ignorant, thus they do not notice potential irritation. The gold line depicts the evolution of the investor irritation weight during the interaction (its value at the *start* of the relevant round is shown). For A;B the simulated investor/trustee had *k* = 2/1, *ζ* = 0.5/0, *α* = 0.4, *P* = 4, $\beta =\frac{1}{3}$. C) Average Investment profiles regenerated from estimated parameters in the full model. All errorbars are standard error of the mean. D) Average Repayment profiles regenerated from estimated parameters in the full model. All errorbars are standard error of the mean. E) Reproduction of sample investor trajectory using 200 simulated interactions with the best fitting parameters. Shaded areas are estimated standard deviation. F) Reproduction of sample trustee trajectory using 200 simulated interactions with the best fitting parameters. Shaded areas are estimated standard deviation.

A detailed example of the general workings of irritation in the case of a single trajectory with potentially aware participants (*q*^*I*^(*ζ*^*T*^) = *q*^*T*^(*ζ*^*I*^) = 2) is shown in Fig 2A. The golden line depicts the evolution of the irritation weight $v_{\iota }^{I}$. At step 2, a subpar repayment by the trustee was introduced by fiat to irritate the investor (the expected repayment by the trustee would have been 50%). The investor’s irritation duly rose to $v_{\iota }^{I}=0.5$. At this point the trustee’s belief about the investor’s irritability is still at 0.5, as they have not observed the investor’s response to their action. At step 3 the investor retaliated against the earlier defection of the trustee. The aware trustee thus updated their irritation beliefs, inferring that the investor was more likely to be irritable (at a marginal probability of *p* = 0.58). Noting the potential cost to the interaction of further irritating the investor, the trustee ensured a better than expected response in the next interaction at step 4. Not only did the trustee repair the interaction, they also ensured that they did not further irritate the investor, at least until the very end of the interaction, as can be seen in the remainder of Fig 2A, from step 4. This exactly captures the “coaxing”-type repair mechanism that King-Casas et al. suggested to explain differences in investment behaviours elicited by healthy control and BPD trustees.

Fig 2B shows the consequence of a lack of irritation inference in the presence of an irritable investor. The players had the same parameter values as in Fig 2A, except for being irritability ignorant (*q*^*I*^(*ζ*^*T*^) = *q*^*T*^(*ζ*^*I*^) = 0). After the same two initial actions (again introduced by fiat), without a notion of the partner being irritable, the trustee missed the chance to repair the interaction at step 3 and the investor’s irritability weight rose to $v_{\iota }^{I}=1$. From this point on the investments stayed low and the trustee did not placate the investor, thus receiving only a paltry total income. Both players failed to extract anything like the full return available from the experimenter.

Quantitative effects of irritability on the group level can be found in the supplementary material S1 Text “Quantitative Illustration of Irritability”.

Fig 2C and 2D show that including these various features removes the discrepancies between data generated from the full model and the subject data. There is no longer a significant difference between generated and original investments or repayments. The complete model predicts 43% of the investor choices (chance is 20%) or equivalently an average NLL of 8.4 on 10 investor choices (from 9.68) and an average NLL of 7.6 or 47% of choice predicted for trustee choices (from 9.5). The richer model is accepted in a likelihood ratio test at a threshold of *p* = 0.006 on the investor side and *p* < 10^−12^ on the trustee side. The final average BIC for the investors is 20.05 and for the trustees is 18.5. A BIC based comparison of all models in this work can be found in the supplementary material (S1 Text “Model Selection”).

Fig 2E and 2F demonstrates that the model qualitatively captures ruptures and repair occurring in real interactions, with the investment decreasing to 0 on 43% and 53% of the sample runs on trials 4 and 7 respectively. Further, the spread of the predictions is greatly reduced from those in Fig 2E and 2F. The investor NLL of this interaction improves from 7.4 to 5.4, while the trustee improves from NLL 11.6 to an NLL of 10.3 (with *ζ*^*I*^ = 0.5; *ζ*^*T*^ = 1).

### Behavioural analysis

The main intent of refining the model was to use it to make inferences about the two investor and two trustee groups that generated the data. In the supplemental material S1 Text “Parameter Recoverability”, we show that such inferences are legitimate in that the parameters are broadly identifiable in self-generated data.

Our prior hypothesis was that either irritability or irritation inferrence would show a significant difference between controls subjects and BPD subjects. This is revealed to be the case, in the form of an irritation belief difference. Additionally, we explored whether previous differences in investor planning and trustee guilt, reported in earlier work [10], would be reproduced in the new model. This turned out to be true for trustee guilt, while the investor planning difference is no longer significant. We then derived an hypothesis that characterized the difference between the two groups, at a level of significance that survived correction for the multiple comparisons undertaken in the derivation of the hypothesis.

**Fig 3:**
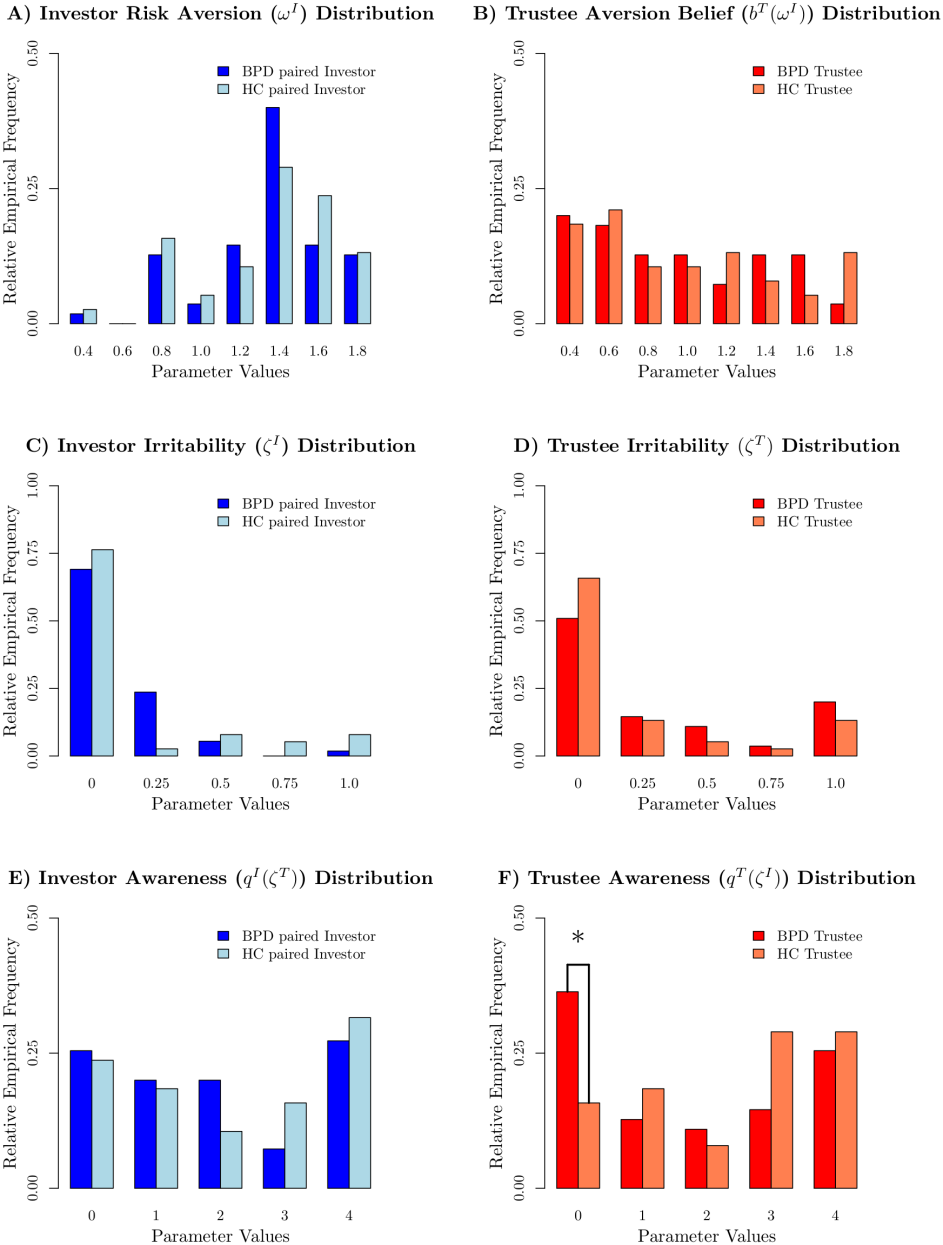
Distributions of newly introduced parameters by group. A) Risk Aversion distribution of investors BPD and HC. B) Risk Aversion distribution of trustees BPD and HC. C) Irritability distribution of investors BPD and HC. D) Irritability distribution of trustees BPD and HC. E) Awareness distribution of investors BPD and HC. F) Awareness distribution of trustees BPD and HC.

The distributions of the new parameters (risk aversion, irritability, awareness) across the groups are shown in Fig 3A–3F. We extend a finding reported in earlier work (see [10]), namely that even in the extended model, the average guilt in BPD trustees is significantly lower in BPD trustees compared to matched (in IQ and socio-economic status) healthy controls (*p* = 0.04, *α*^*T*^: 0.32 < 0.49, uncorrected for multiple comparisons). This can be traced back to a significantly higher proportion of guilt *α*^*T*^ = 0 subjects (*p* = 0.02, *χ*^2^-test for equal proportions, uncorrected for multiple comparisons). Additionally, the irritation ignorant awareness setting (*q*^*T*^(*ζ*) = 0) is significantly more common in BPD trustees, compared to HC trustees (*p* = 0.03, *χ*^2^-test for equal proportions, uncorrected for multiple comparisons).

We therefore considered a model-based characterization of the subjects in which we combined together the two key differences between HC and BPD trustees in the model: trustees who are either totally guilt-less (*α*^*T*^ = 0) or who are irritation unaware (*q*^*T*^(*ζ*) = 0), or both. Either of these leads to trustees who may exploit the investor (deliberately for *α*^*T*^ = 0 or accidentaly at *q*^*T*^(*ζ*) = 0), and so create problems in the context of an interaction in which latter is in charge. We describe these trustees as being ‘perilous’.

This combined group turns out to be present at a significantly higher proportion (60.0%) in the BPD group, compared with the HC group (29%) (*p* = 0.003, *χ*^2^-test for equal proportions). The difference remains significant (*p* < 0.05) even when Bonferroni correcting for the 7 (4 parameters plus the 2 proportion tests and the derived “perilous group” hypothesis) comparisons that we undertook.

**Fig 4:**
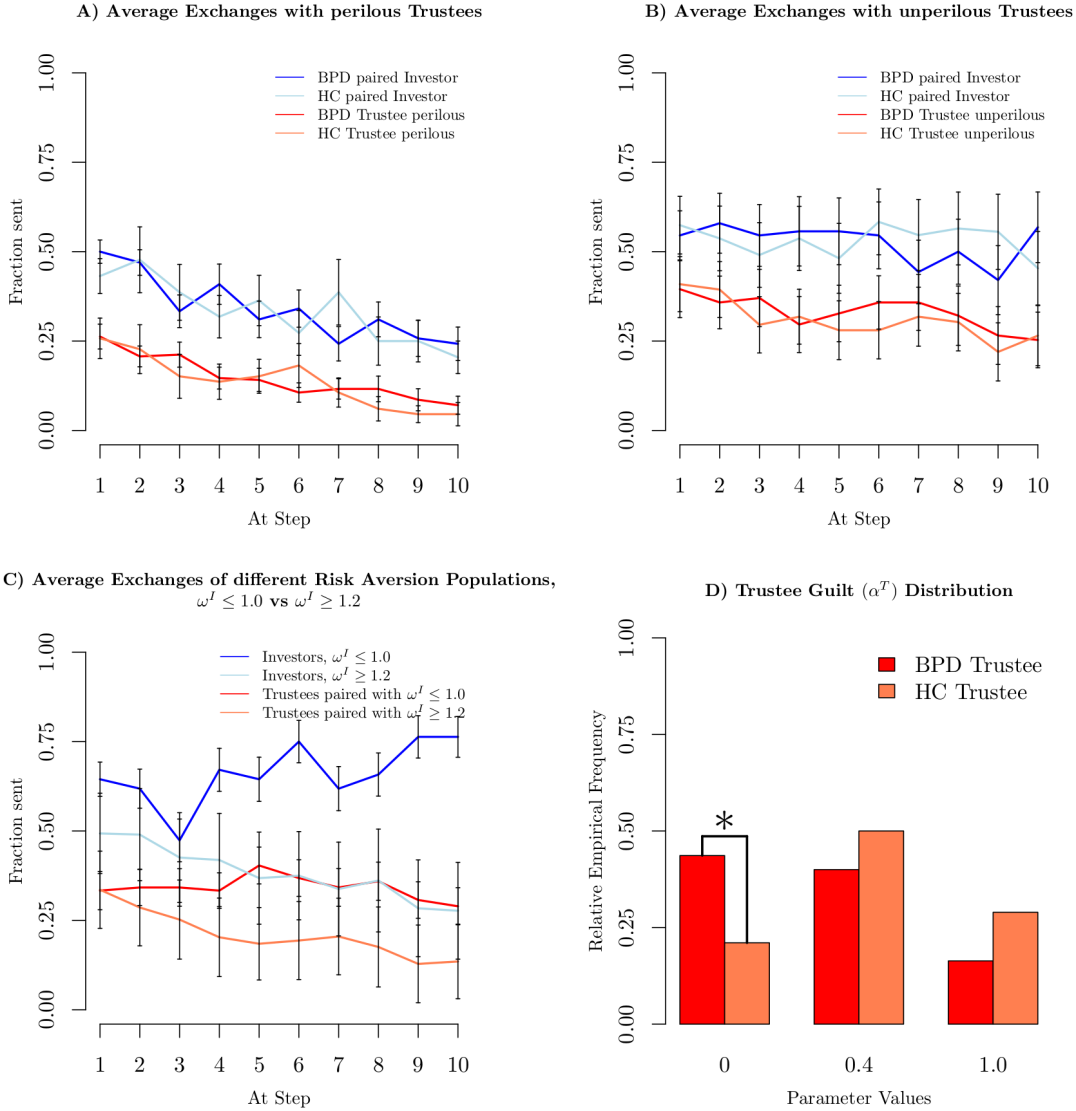
Model based data features. A) Investment and return profile for subgroups of the BPD and HC data sets, defined by *ζ*^*T*^ > 0 or *q*^*T*^(*ζ*) = 0. B) Investment and return profile for subgroups of the BPD and HC data sets, defined by *ζ*^*T*^ = 0 and *q*^*T*^(*ζ*) > 0. C) Investment and return profile for subgroups defined by *ω*^*I*^ ≤ 1.0 (blue, red) or *ω*^*I*^ ≥ 1.2 (light blue, coral). D) Guilt distribution of trustees BPD and HC. All errorbars are standard error of the mean.

Fig 4 shows investment and repayment profiles for dyads in our data set including perilous (A) and non-perilous (B) trustees. These interaction profiles are evidently different (confirmed in two-sided t-tests at *p* < 0.05, Bonferroni corrected for the 10 time points). Yet, having adjusted for this by sorting healthy controls and BPD trustees according to perilousness, there is no longer a difference between the average investment and return profiles for BPD versus HC dyads (*p* > 0.05 using an uncorrected two-sided t-test).

Fig 4C compares investment and return profiles for investors with little (*ω*^*I*^ ≤ 1.0) or substantial risk aversion (*ω*^*I*^ > 1.0). Splits based on trustee risk aversion profiles *b*^*T*^(*ω*^*I*^) do not appear significantly different (which is also a testament to the dominant role of the investor) and are not shown here. Finally, Fig 4D shows the distributions over the guilt parameters for BPD and HC trustee subjects.

## Materials and methods

### Ethics statement

Informed consent was obtained for all research involving human participants, and all clinical investigation was conducted according to the principles expressed in the Declaration of Helsinki. The procedures were approved by the Institutional Board of Baylor College of Medicine.

### Technical data

Programs were run at the local Wellcome Trust Center for Neuroimaging (WTCN) cluster using Intel Xeon E312xx (Sandy Bridge) processor cores clocked at 2.2 GHz; no process used more than 0.8 GB of RAM. We used R [26] and Matlab [27] for data analysis and the boost C++ libraries [28] for code generation.

### Algorithmic change

Our earlier approach (in [10]) utilized a sampling based method to explore the decision tree during planning in the trust game, drawing from approximate solution methods for tree search from machine learning (see [29–32]). However, if lower levels of calculation are all part of the same hierarchy, as well as kept in memory and so are immediately available for higher level calculations, then the problem scales linearly in the theory of mind level parameter, rather than exponentially (as for other computational approaches to I-POMDPs, [33], p.325, 9.2.). This trade off of memory for computation is only practical if the planning horizon is reduced to at most 4 steps into the future. A more detailed discussion of the used algorithm can be found in the supplementary material S1 Text “Algorithmic Representation”.

The net result is that it takes less than 2 minutes per generated 10 step interaction, to calculate deterministically (i.e., avoiding approximations from the stochasticity of Monte Carlo-based tree evaluation) a 10 step exchange of a level *k*^*I*^ = 4 investor with a level *k*^*T*^ = 3 trustee, both having horizons of *P* = 4 steps. This comes at the cost of having to commit 0.8 Gb of RAM to the tree calculation.

### Earlier and related work

Trust games of various kinds have been used in behavioural economics and psychology research (see [34]). In particular, the MRT we used was based on variants in several earlier studies (see examples in [17, 35, 36]).

The current MRT was first modeled using regression models (see [16]) of various depths: one step models for the increase/decrease of the amount sent to the partner and models which track the effects of more distant investments/repayments. These models generated signals of increases and decreases in investments and returns that were correlated with fMRI data. One seminal study on the effect of BPD in the trustgame by King-Casas et al. (see [6]) included the concept of “coaxing” (repaying substanially more than the fair split) the partner (back) into cooperating/trust whenever trust was running low, as signified by small investments.

Furthermore, an earlier study (see [37]) used clustering to associate trustgame investment and repayment levels to various clinical populations.

An I-POMDP generative model for the trust task which included inequality aversion, inference and theory of mind level was previously proposed [8]. This model was later refined rather substantially to include faster calculation and planning as a parameter [10].

The I-POMDP framework itself has been used in a considerable number of studies. Notable among these are investigations of the depth of tactical reasoning directly in competitive games (see [38–40]). It has also been used for deriving optimal strategies in repeated games (see [41]).

The benefits of a variant of the framework for fitting human behavioural data were recently exhibited in [42].

## Discussion

Our previous model of the complex collections of choices apparent in the multiround trust task did a generally good job at accounting for many aspects, and generated prediction errors and other parametric regressors that unearthed various key neural processes. However, on closer inspection, it failed to characterize aspects of behaviour at two disparate timescales: a persistent reluctance of the dominant party to submit a portion of their endowment to the potentially fickle trustee in the game; and temporary breakdowns in cooperation and consequent repair. We therefore enriched our model in these two respects, parameterizing risk aversion (a factor that had previously been suggested as potentially corrupting the measurement of trust with this task, see [12]), and irritation.

There is a subtlety in the differentiation between risk aversion and trust. In our formulation, risk aversion is a parameterized quantity providing an intrinsic limit to how valuable a potential, yet uncertain, repayment is to the agent. By contrast, trust is not directly parameterized; rather, it is an emergent consequence of the evolving interaction between the two players. However, if one attempted to measure the degree of an investor’s trust by the amount invested with the trustee, then risk aversion would be an expression of a lack of trust. We continue to distinguish the quantities, since trust evolves dynamically through inference about guilt and irritation; whereas risk aversion is ultimately fixed.

Despite its formal appeal, it is challenging to use the I-POMDP model to characterize game theoretic interactions between players. One obvious reason for this is its apparent computational cost. Here we showed that it is perfectly possible to perform approximate I-POMDP inference in a relatively complicated model with two intentional dimensions and various other parameters. This augurs well for the future, given the importance and richness of social interactions in both economic decision-making, and as a psychological biomarker in psychiatric conditions.

Our extension of the I-POMDP framework to allow internal state shifts (and agents that may be aware of such shifts) adds a crucial layer of flexibility to these approaches. We illustrated this using irritation as an elemental emotional process. This captured the rupture and repair of cooperation, along with the associated threats of these. In the same way that the possibility of punishment or defection maintains cooperative behaviour in tasks such as the public goods game, the possibility of rupture encourages healthy participants to be beneficent. We hope that similar mechanisms will also be useful to describe strategic interactions in other tasks. We will also use it to guide the analysis of functional brain imaging data.

This model of irritation departs from conventional models of intentional inference in one important way. In repeated social exchange tasks, it is conventional to model one’s partner’s *preferences*, which, in Bayes-Nash terms, concerns properties of their *utility functions*. Indeed, this is exactly how earlier studies on the multi round trust game framed the social exchange (see [8–10, 37]). Here, however, we considered simultaneous intentional inference about both a *utility* and a *policy* (as in [41]) that the player would adopt (indeed, a policy that it would be hard to justify in pure utility terms, given the costs of breaking cooperation). We include the possibility of one agent’s actions changing the *intentional state* of another agent, thus extending beyond the non manipulability assumption in the original I-POMDP work (see [1]) and providing a tracktable time series of irritation/state shifts (see Fig 2A and 2B). This non-stationarity could be accommodated within the parameterized I-POMPDs of Wunder et al. (see [41]), using a specially-fashioned extension of the intentional state space.

A richer palette of such internal state-shifting default behaviors might also prove important in other tasks. Note, though, that it is not yet clear that a suitable notion of equilibrium can be defined (for instance, as the theory of mind level of the players tends to infinity). The combination of Kuhn’s theorem (see [43]) (since our players have perfect recall) and Harsanyi’s treatment of mixed strategies (see [44]) might be a starting point for such a treatment.

Our approach to irritability was chosen for its simplicity within the existing model. Further work on a more substantial body of human data will be necessary to fine-tune the dynamics of irritation in social exchange. One first step might be to use the model as part of an optimal experimental design framework to realize a computer-based opponent that could extract the most out of each available choice. At present, the relatively small number of actions in our version of the trust task, together with the possibility that the human partners fail to irritate each other even when they are irritable, leaves little room for further sophistication. Given a better understanding of irritation in the model, it would then be possible to refine the concept itself.

The ultimate model has the uncomfortable characteristic of employing 7 parameters to account for the 10 choices of each subject. However, the parameters interact in complex ways in the model, which is why they can generally be reliably inferred, as apparent in the confusion matrices in the Supplementary materials.

Finally, the model provides a generative approach to the way that patients with Borderline Personality Disorder play in the multi round trustgame, as reported in King-Casas et al. This approach yields a particular type of trustee, who are perilous for the interaction; this type was overrepresented in the BPD sample. After taking proper account of this subtype, we found equal average behaviours in BPDs and HCs. Thus this subgroup (which is also present in the HC group, albeit to a lesser extent) could help separate out a clinical phenotype that is separate from those sufferers of BPD who are less susceptible to the breakdown of trust. Such a separation might yield clearer clinical and neurological characterisations. It would be most interesting to look for, and analyze the clinical correlates of, types analagous to perilousness for players who are in control of interactions, like the investors here.

### Limitations

Although we provided an additional characterisation of the difference between the healthy and clinical populations studied in [6], the psychiatric validity of our model parameters has yet to be established. In particular, we lacked additional clinical scales and personality measures for these populations; we are presently collecting new data that will allow a more comprehensive assessment. In section S1 Text “Predictive Validity through Comparison with other Games”, we provide a reason to expect the inferred parameters to characterize something generalizable about a different group of subjects by showing how they relate to parameters derived from a model of subjects’ performance of the ultimatum game.

In addition, the notion of perilousness and its effects have been derived post hoc on the well studied data of earlier works [6]. We are working to test on newly collected data, whether this notion and its effects can be reproduced.

In terms of parameter identifiability, we illustrate the internal consistency of the model in section S1 Text “Parameter Recoverability”. When looking at section S1 Text “Model Selection”, we see that the correct model is identified on generated data on the group level in almost all cases, the exception being the difference between trustee data generated from the original model, being estimated to have come from the model with variable temperature and risk aversion.

Computationally, the costs of planning limit our exact calculation to a planning horizon of 4. While we consider this sufficient, as we were able to reproduce all behaviours seen at a planning horizon of 7 in our earlier work, some more complex behaviour may have been eliminated through the restricted planning horizon.

Finally, computational limitations force us to use a coarser representation of the MRT than might be optimal, both in terms of representing possible subject actions and in terms of the number of discrete parameter settings.

## Supporting information

S1 Text Summarized supplementary material. Details on various more computational aspects of the model and its implementation. It is divided into chapters: “Theory of Mind Limitation”, “Planning”, “Model Selection”, “Risk Aversion”, “Quantitative Illustration of Irritability”, “Parameter Recoverability”, “Algorithmic Representation” and “Predicitve Validty through Comparison with other Games”.
(PDF)
