# Supplementary material for: A model of risk and mental state shifts during social interaction
Source: PLoS Comput Biol. 2018 Feb 15;14(2):e1005935. doi: 10.1371/journal.pcbi.1005935 (PMC5831643; doi:10.1371/journal.pcbi.1005935)
Supplement: S1 Text — Details on various more computational aspects of the model and its implementation. It is divided into chapters: “Theory of Mind Limitation”, “Planning”, “Model Selection”, “Risk Aversion”, “Quantitative Illustration of Irritability”, “Parameter Recoverability”, “Algorithmic Representation” and “Predicitve Validty through Comparison with other Games”. (PDF) [file pcbi.1005935.s001.pdf]

S S1 Text

Theory of Mind Limitation

In Fig S1, we display average interactions of investor and trustees, with basic parameters: $\alpha^I = \alpha^T = 0.4$, $P^I = P^T = 4$, $\beta^I = \beta^T = \frac{1}{2}$ and various theory of mind levels. Investors and Trustees are always one level apart. In the left column the investor has the higher level and in the right column the trustee has the higher level. Since their guilt is 0.4 the trustee will try to exploit the investor. In the left column the investor is of a higher level and therefore sees through the trustee's deception and lowers the investment. In the right column the trustee is of higher level and successfully exploits the investors. From the level 4 investor (Fig S1C)) the behaviours do not appear to be qualitatively different anymore, except for minor differences in rates of increase of decline, which can be accounted for by the new parameter of risk aversion and inverse temperature (see the main text). Therefore we restricted our data fit to a maximum ToM level of 4.

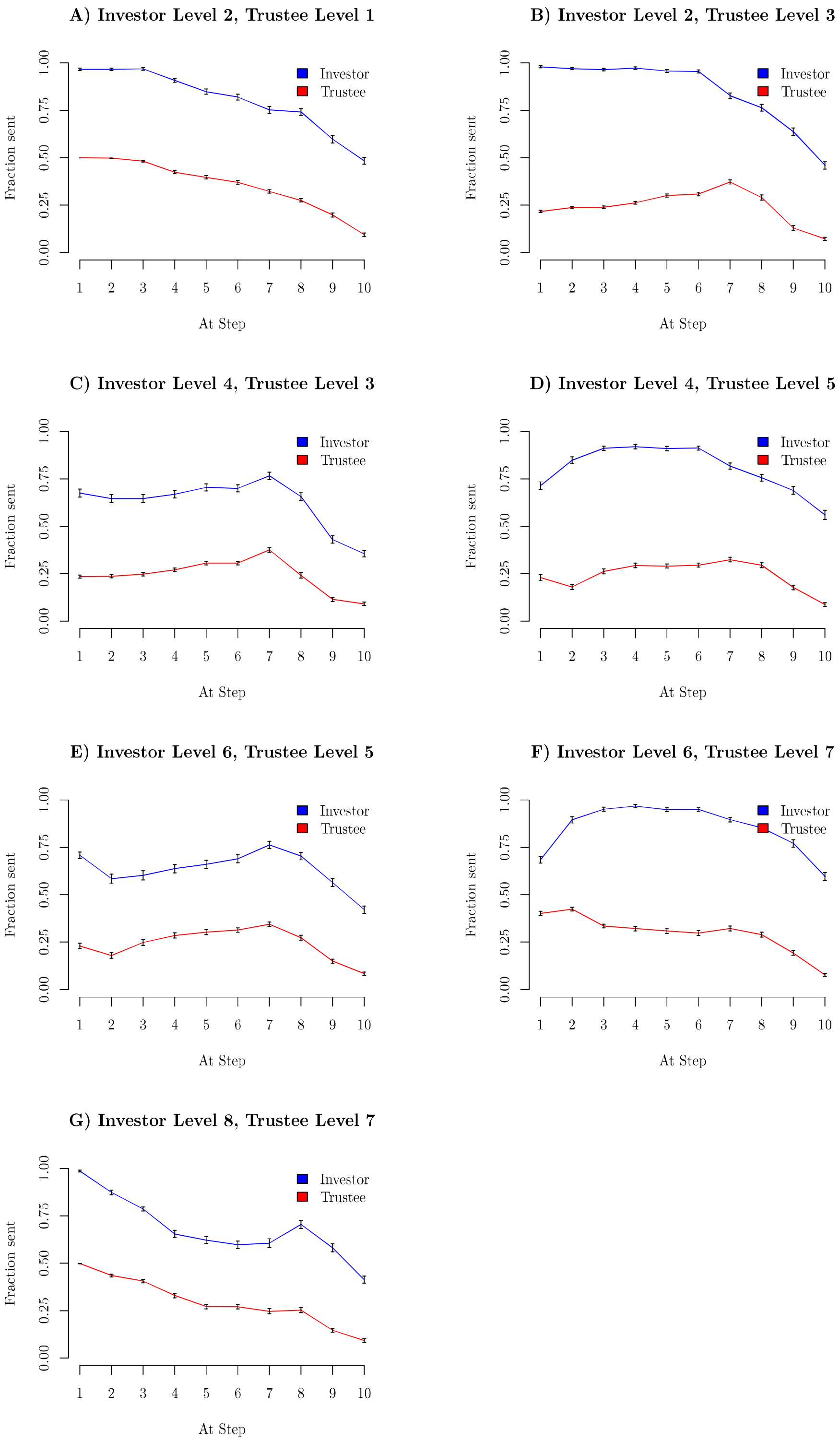

Fig. S1: Averages of 200 sample paths, displaying interactions between investors and trustees one level apart, with parameters $\alpha^I = \alpha^T = 0.4$, $P^I = P^T = 4$, $\beta^I = \beta^T = \frac{1}{3}$. left column: Investor Level is higher. Right column: Trustee Level is higher. All errorbars are standard error of the mean.

Planning

In this section, we confirm that we can recover the paradigmatic behaviours of our earlier work (see [10]) with planning 4 as well as we could with planning 7 in the earlier work. The essential behaviours can be seen in Fig S2A-D. Fig S2A is based on a level 2 investor and a level 1 trustee, with $\alpha^I = \alpha^T = 0.4$ and $\beta^I = \beta^T = \frac{1}{3}$ and $P^I = P^T = 4$: The trustee tries to build up trust and then exploit the investor. The investor being level 2 is not deceived by the trustee and reduces their investment, as the trustee defects, thus *preempting exploitation*. Conversely in Fig S2B, the investor is level 0 and $\beta^I = \beta^T = \frac{1}{3}$, with the other parameters being the same. In this case, the trusee successfully builds trust in the first few exchanges and later on exploits the investor, who keeps giving till the very last exchanges, believing the trustee to trustworthy. In Fig S2C the "impulsive" behaviour is repoduced: It shows the average exchanges of a level 0 investor and a level 1 trustee, with $\alpha^I = \alpha^T = 0.4$ and $\beta^I = \beta^T = \frac{1}{3}$ and $P^I = P^T = 2$. The planning horizon leads the trustee to exploit the investor too early, therefore earning much less than in the case of Fig S2B, despite being also of higher level than the investor and having a matched planning horizon. Finally, Fig S2D demonstrates the importance of the planning horizon by showing average exchanges of a level 2 investor and a level 1 trustee, with $\alpha^I = \alpha^T = 0.4$ and $\beta^I = \beta^T = \frac{1}{3}$ and $P^I = 2$ but $P^T = 4$: Although the investor is of a higher level, the longer planning/more consistent trustee successfully deceives them, with the light drop off in investments in the end being more due to horizon effects than to the investor looking through the trustee's deception.

We do not present the fully cooperative (guilt 1) case as well as the fully greedy case (both partners guilt 0) since those are essentially unrelated to depth of planning.

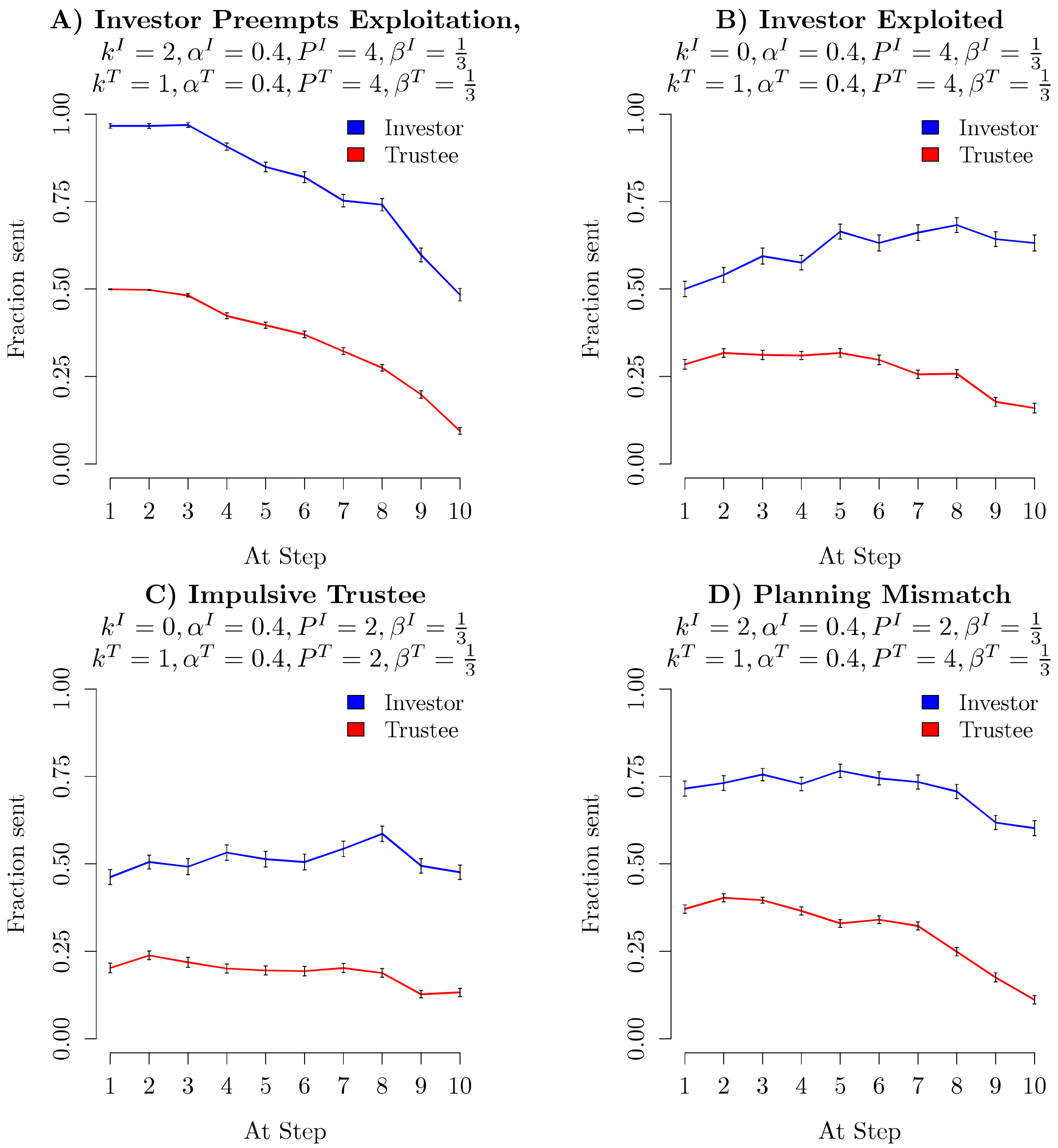

Fig. S2: Averages of 200 sample paths. A) Investor reacts to exploitation by trustee . B) Investor is exploited by trustee . C) Trustee is too inconsistent to exploit Investor D) Trustee exploits higher level shorter planning Investor. All errorbars are standard error of the mean.

In the face of these reproductions, we can conclude that all paradigmatic behaviours of our earlier work can just as well be produced with planning 4 as with planning 7.

Model Selection

We confirm the improvements of the old model by means of likelihood ratio tests of each model parameter against the model without that parameter added. Additionally, The BIC for the diverse submodels can be found in table S1.

Table of Model Selection Criteria

Model Parameter Set θ	p Inv	p Tru	BIC Inv	BIC Tru
(α, P, k)	$-$	$-$	2534	2232
(α, P, k, β)	$< 10^{-15}$ *	$< 10^{-15}$ *	2320	2038
$(\alpha, P, k, \beta, \omega)$	$< 10^{-15}$ *	$-$	2016	$-$
$(\alpha, P, k, \beta, b(\omega))$	$-$	$0.3\ X$	$-$	1982
$(\alpha, P, k, \beta, \omega, b(\omega), \zeta)$	$0.12\ X$	$> 0.9\ X$	1948	1962
$(\alpha, P, k, \beta, \omega, b(\omega), \zeta, q(\zeta))$	0.01 *	$< 10^{-15}$ *	1864	1722

Table S1: Likelihood ratio p-values for the acceptance of the simpler model (with the last parameter in θ removed) compared to the more complex model θ. Rejections of the simpler models at a probability $p < 0.05$ are marked with an asterisk, instances of acceptance of the simpler model are marked with "X". Data sets for which there is no difference between the richer and the next simpler model are marked by "$-$". "Inv" denotes the "investor" side likelihood. "Tru" denotes the "trustee" side likelihood. Sum BIC scores for investor and trustee Data, for the nested models in this work. The top most model corresponds to the one used in [10]. The next model includes the inverse temperature parameter β. The following two models include risk aversion on the investor side ω and the risk aversion belief on the trustee side $b(\omega)$. Finally, the last two models include irritability ζ and irritation inference respectively $q(\zeta)$.

This shows that the models in the main text are justified in terms of likelihood ratio tests and BIC improvements, either on the investor or the trustee side. Noticeably, the "intermediary" step of adding just irritability would be rejected, but in conjunction with irritation inference it is accepted. We add that, as stated in the main text, the direct likelihood ratio test between the model with risk aversion and the model with both irritation and irritation inferrence decides in favour of the latter. Similary, adding the risk aversion belief to the trustee would not pass a likelihood ratio test criterion, but we allow the trustee to assume investor risk aversion nevertheless, for reasons of model consistency.

Furthermore, we want to test whether we can accurately determine which model surrogate data was generated from. We generated data according to all 3 "main" models, using the estimated parameters of the $n = 93$ subjects of the main text data set. We then ran parameter estimations on the trajectories generated from all 3 models. The likelihood ratio test p-values against the next simpler model on each data set can be seen in table S2.

Likelihood ratio tests for Surrogate Data of the 3 models

Model Label	Data Generated from	p Inv M_2	p Inv M_3	p Tru M_2	p Tru M_3
M_1	(α, P, k)	$0.25\ X$	$> 0.9\ X$	10^{-5} * !	$> 0.9\ X$
M_2	$(\alpha, P, k, \beta, \omega, b(\omega))$	$< 10^{-15}$ *	$> 0.9\ X$	10^{-12} *	$> 0.9\ X$
M_3	$(\alpha, P, k, \beta, \omega, b(\omega), \zeta, q(\zeta))$	$< 10^{-15}$ *	$< 10^{-15}$ *	$< 10^{-5}$ *	$< 10^{-15}$ *

Table S2: p-values of likelihood ratio test results for accepting model M_{i-1} compared to M_i (specified in the column), for the 3 main models on generated trajectories ($n = 93$) from same models (specified in the rows). Rejections of the simpler models at a probability $p < 0.05$ are marked with an asterisk, instances of acceptance of the simpler model are marked with "X". Acceptance of a more complicated model than the data was generated from is specified by "!". "Inv" denotes the "investor" side likelihood. "Tru" denotes the "trustee" side likelihood.

As we can see, the correct model wins in almost all cases. The single exception being trustees generated from the original model, which could be missclassified to be generated from the model augmented by a variable inverse temperature and risk aversion (belief).

Risk Aversion

The effect of risk aversion in shifting investment levels can be seen in figures S3A;B. These depict the average investment (A) and repayment (B) trajectories over 200 simulated exchanges in which a trustee with $b^T(\omega^I) = 1$ (i.e., who believes the investor to have $\omega^I = 1$) interacts with investors of varying actual ω^I values (other parameters are given in the caption). Cooperative trustee actions early in the game can make the investor overcome moderate levels of risk aversion (the curve for $\omega^I = 1.2$ merges with the curve for $\omega^I = 0.8$ in the early trials). Higher risk aversion levels ($\omega^I = 1.4$) delay the positive effects of cooperation, such that it increases from a low initial level until step 6, but then drops abruptly due to horizon effects and trustee defection. For the highest risk aversion levels ($\omega^I = 1.6; 1.8$) in Fig S3A, inference about other parameters may be hampered. That is, if investments stay low throughout, risk aversion might become nearly the only parameter that can be inferred with certainty. This implies a constraint on any further statistical treatment of behavioural data or derived quantities, such as model-based fMRI analysis.

Fig S3C depicts the effect of the risk aversion belief on the trustee, with the investor now being fixed at $\omega^I = 1.0$ (and not shown). For $b^T(\omega^I) < 1.0$, it can be seen that trustees make early attempts to exploit investors they think are not risk averse, since they assume the investor is still more likely to invest in them than to defect. However, they then defect rather quickly. As the trustee's belief approaches the utilitarian setting $b^T(\omega^I) = 1.0$, she becomes more cooperative and for a longer time period into the game, since this is necessary to keep the investments of the utilitarian investor going. Given a high setting of $b^T(\omega^I) = 1.4$ the trustee returns little, as they consider the likelihood of the investor to keep investing in them to be low and thus see no gain from building up cooperation.

As Fig S3D;E demonstrate, the investment can on average be well reproduced after including risk aversion in the model, although there remains a significant under-reciprocation on the part of the generated HC trustee, compared with the real exchange data. Further, Fig S3FG demonstrates that despite an improvement in fit (NLL for the investor goes from 14.04 to 7.47 with $\omega^I = 1.4$; for trustee the NLL goes from 11.93 to 11.53 with $b^T(\omega^I) = 0.6$), this model remains incapable of capturing the transient rupture and - by extension - repair that we examined in Fig 2D. Again, the modelled investor decreased their investment to 0 on only 23% of the sample runs on trials 4 and 7, compared with the collapse in the actual investment.

Quantitative Illustration of Irritability

Fig S4 shows the effect of irritability and irritability inference on average behaviours over 200 simulated exchanges. As is also evident in Fig 2, these averages blur the precise times at which the ruptures happen, but show the consequences in terms of net cooperation. In both cases (A-C; D-F), the trustee is more sophisticated than the investor ($k^I = 0; k^T = 1$) , the investor is either irritable or non-irritable, but unaware; the trustee is nonirritable (other parameters are listed in the caption). The difference between the figures is that the trustee is aware in Fig S4A-C, but unaware in Fig S4D-F.

Fig S4A show average investment and returns when the trustee is aware for an irritable (dark) and non-irritable (light) investor. The trustee's awareness enables her to keep the investment at almost the same level in both cases. This arises from the excess return that she provides. Thus, as in Fig 2F, trustees who realize that their investors are irritable will delay exploitation to the late rounds of the game. For the case of the irritable investor, Fig S4B shows the average evolution of the inference about partner irritability. The trustee becomes aware that their partner is likely irritable after the first retaliation (as in the particular case in Fig 2F). The average internal state of the same investor and trustee can be seen in Fig S4C. Overall the irritation weight of the investor is kept low by an aware trustee, who can repair the interaction if needed. The value of irritability between investor and trustee is not symmetric in this example, so that the trustee may reliably repair or fail

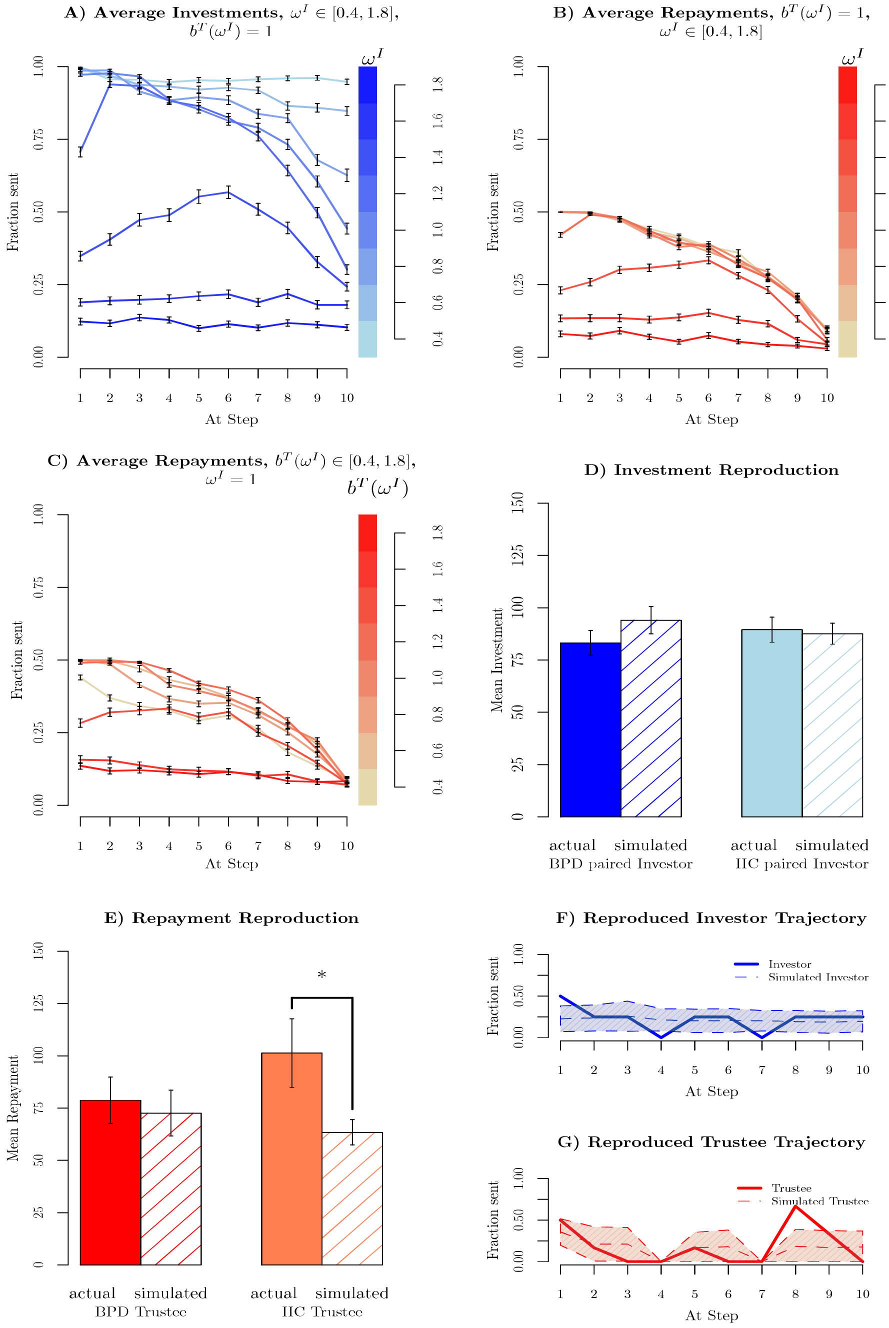

Fig. S3: A) Investment profiles for different settings of investor risk aversion. All errorbars are standard errors of the mean. Here, the investor and trustee have guilt $\alpha = 0.4$, inverse temperature $\beta = \frac{1}{2}$, and planning $P = 4$; the investor (respectively trustee) has ToM level $k^I = 2$ ($k^T = 1$). B) Trustee repayment profiles for the interactions depicted in A. C) Repayment profiles for different settings of trustee risk aversion belief. All errorbars are standard errors of the mean. Here, the investor and trustee have guilt $\alpha = 0.4$, inverse temperature $\beta = \frac{1}{2}$, and planning $P = 4$; the investor (respectively trustee) has ToM level $k^I = 2$ ($k^T = 1$). D) Average Investment profiles regenerated from estimated parameters. All errorbars are standard error of the mean. E) Average Repayment profiles regenerated from estimated parameters. All errorbars are standard error of the mean. An asterisk denotes a significant difference ($p < 0.05$, two sided t-test) in means between the original data and the generated exchanges. F) Sample investor trajectory vs average of 200 generated exchanges using the model augmented by risk aversion. Shaded areas are estimated standard deviation. G) Sample trustee trajectory vs average of 200 generated exchanges using the model augmented by risk aversion. Shaded areas are estimated standard deviation.

to repair without being themselves subjected to the effects of irritation. We note that the investor is driven up to near equally high investments in the non-irritable case in Fig S4A compared to that in the irritable case of Fig S4A, despite the trustee actually returning less. This is because the level $k^T = 1, q^T(\zeta^I) = 0$ trustee knows exactly what actions they need to take in order to confuse the inference of the level $k^I = 0$ investor (i.e. what responses the investor will consider unlikely). By contrast, the level $k^T = 1, q^T(\zeta^I) = 1$ trustee in Fig S4A accounts for potential irritability right away and thus has to "play along" with the investor $k^I = 0$'s expectations and is less effective in tricking their inference.

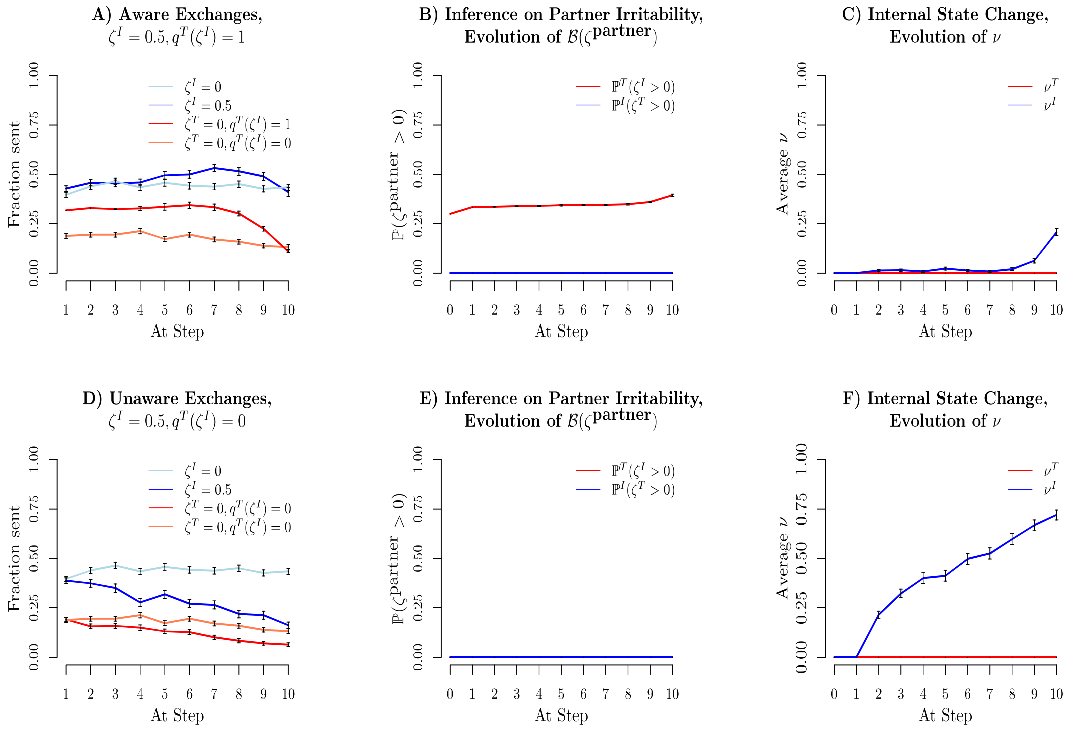

Fig. S4: A-C) Simulated repair interaction with an irritability aware trustee ($q^T(\zeta^I) = 1$) and unaware investors who are irritable (dark lines; $\zeta^I = 0.5$) or non-irritable (light lines; $\zeta^I = 0$). A) Average investment profiles in the two cases. B) Average evolution of the irritability beliefs of both partners. The trustee learns correctly that the irritable investor is irritable. C) Average evolution of the irritation weight v_ι for both partners. The awareness keeps the irritation weight low and the trustee can repair the interaction if needed. D-F) Simulated breaking interaction with the same investors, but an unaware trustee ($q^T(\zeta^I) = 0$). The plots show the same quantities as in A-C. All errorbars are standard errors of the mean over 200 simulations. Investor/trustee parameters are $k = 0/1; \alpha = 0.4; \omega = 1.4; P = 4; \beta = \frac{1}{2}$ and the trustee had the fixed belief that the investor's risk aversion was $\omega = 1.4$.

By contrast, Fig S4D shows that if the trustee is unaware, then there can be ruptures of cooperation that become apparent even at the group average level. The evolution of the beliefs about irritation is nugatory, as can be seen in Fig S4E. Fig S4F shows how the irritation weight reaches high values quickly, only occasionally being reduced by chance repair.

Parameter Recoverability

The ultimate model is rather complicated. This raises the concern that the same behaviour might result from radically different settings of the parameters, implying that we would not be able to draw

stable or meaningful conclusions from fitting behaviour. Indeed, we have already observed that certain settings will make it impossible to make inferences about some parameters – thus, playing with a highly risk averse investor will give no opportunity for a trustee to express her individual characteristics.

To examine this, we assessed parameter recoverability. That is, we used the parameters obtained from ML estimation on the participants (note that not all values were represented in the population – $\omega^I = 0.6$ is absent, for instance), generated new data *ab initio* from the model, fitted the new data, and quantified any discrepancies between the original and recovered parameters. Figures S5 and S6 show the probability of recovering either the actual or a neighboring parameter value for investor and trustee respectively.

It is apparent that the model has some significant purchase on all the parameters. However, some parameters are much harder to estimate than others. There are perhaps four most egregious forms of confusion. First, irritable subjects can be inferred as being non-irritable (figures S5F; S6F). This occurs if the remaining randomness of the interaction in the model is such that the investor's irritation is not excited. Indeed, the task was not designed with irritation in mind, and so players are not forced or encouraged to irritate each other.

Second, the investor's awareness is not very reliably recovered (Fig S5E). It is slightly better recovered for the trustee (Fig S6E), who faces a more stringent challenge to keep the investor trusting them.

Thirdly, the inverse temperatures β^I of generated investor trajectories tend to be overestimated (Fig S5B). This is not surprising since the preferred actions will be the same for several temperature settings, thus requiring several "unlikely" actions to identify a lower β^I in investors.

Finally, there is a tendency to missestimate the risk aversion belief of the trustee, for high settings of $b^T(\omega^I)$, as the parameter apparently has less influence on trustee choice, than it does for the investor, who is in control of the interaction (see Fig S6G). This effect may be driven by the fact that the trustees estimated to have such high beliefs were also estimated to be considerably more irritable and thus the ensuing breaks might have confounded the inference about the risk aversion belief or vice-versa per chance lower repayments could have been interpreted as irritability. Globally, irritability is under rather than overestimated, however we can not rule out a particular interaction for high $b^T(\omega^I)$ subjects.

Also, many parameter values which are less reliably recovered are also the less common values in the estimated data to begin with, thus making their recovery subject to a higher volatility by means of lower numbers.

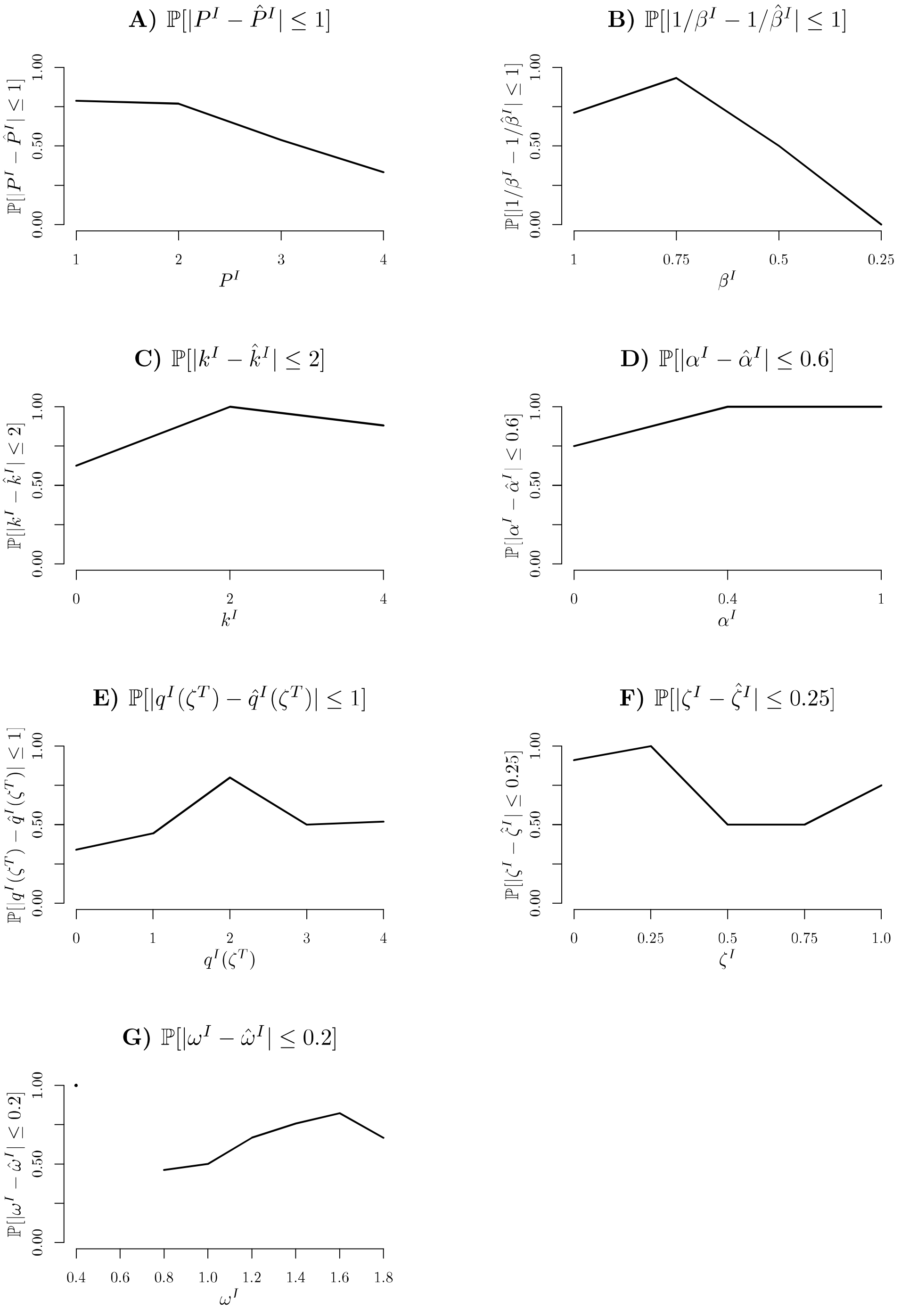

Fig. S5: For the investor: Probability of Parameter recovery or recovery of a neighbouring parameter value from generated exchanges using real subject parameters. A) Probability to recover the planning value P^I or a neighbouring one. B) Probability of recovering β^I or a neighbouring value. C) Probability of recovering ToM k or a neighbouring value. D) Probability of recovering Guilt α^I or a neighbouring value. E) Probability of revovering $q^I(\zeta^T)$ or a neighbouring value. F) Probability of recovering ζ^I or a neighbouring value. G) Probability of recovering risk aversion ω^I or a neighbouring value. The value $\omega^I = 0.6$ did not occur in the original data set.

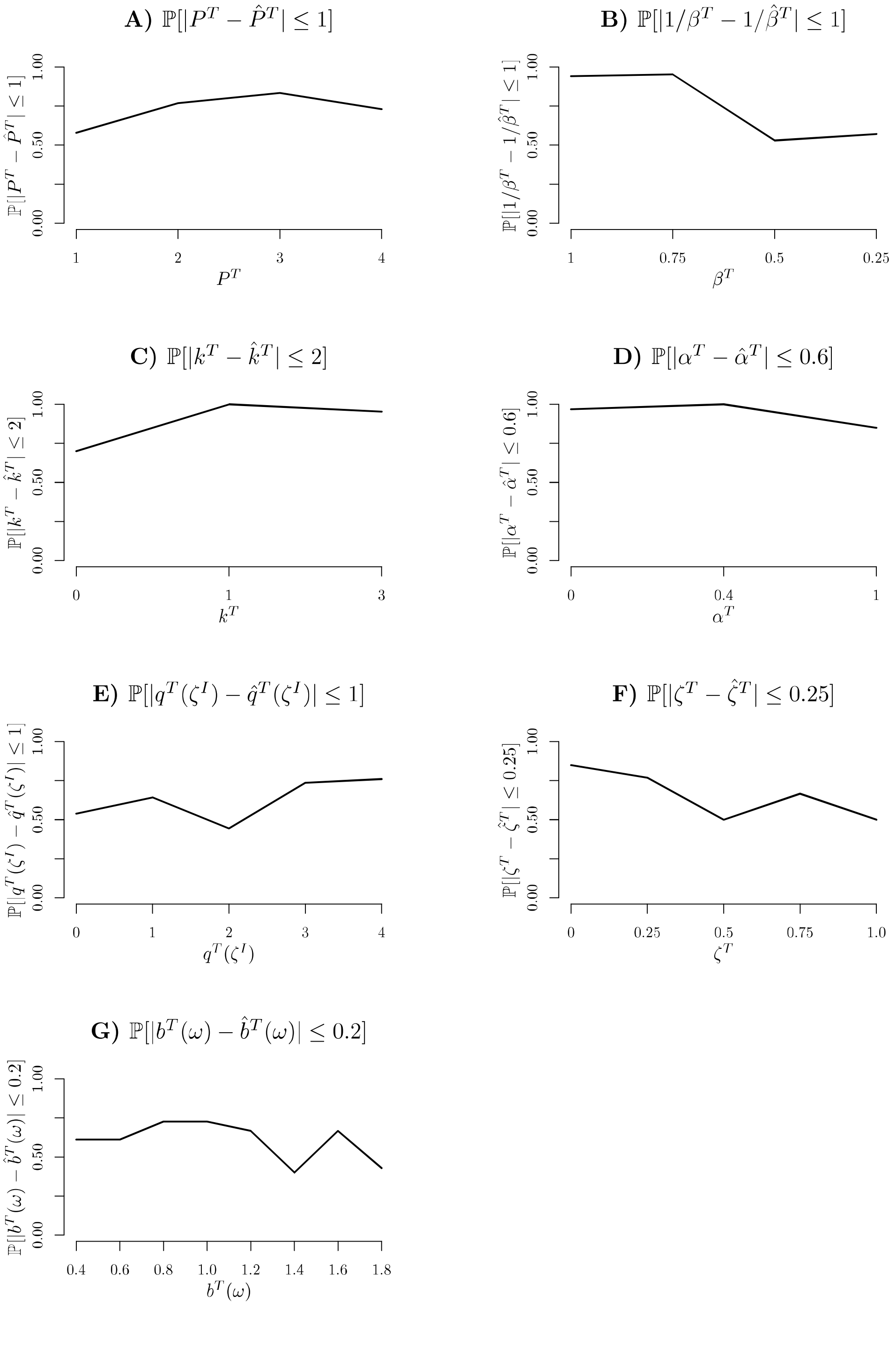

Fig. S6: For the trustee: Probability of Parameter recovery or recovery of a neighbouring parameter value from generated exchanges using real subject parameters. A) Probability to recover the planning value P^T or a neighbouring one. B) Probability of recovering β^T or a neighbouring value. C) Probability of recovering ToM k or a neighbouring value. D) Probability of recovering Guilt α^T or a neighbouring value. E) Probability of revovering $q^T(\zeta^I)$ or a neighbouring value. F) Probability of recovering ζ^T or a neighbouring value. G) Probability of recovering risk aversion $b^T(\omega^I)$ or a neighbouring value.

Algorithmic Representation

We outline the algorithm through which we achieve linear running time in the theory of mind level for the given I-POMDP problem (under no environmental uncertainty i.e. the only source of uncertainty being the future partner actions).

The components used in the calculation are the observation-action history h = $\{o_0, a_0, o_1, a_1, \ldots\}$ (encoding actions taken a and observations made o in temporal sequence, as indicated by a time subindex), the target theory of mind level k, the remaining steps till the time horizon P, the reward expectation E, the material reward r(a,o, θ) of observing o after action a, the probability p(o) of observing o upon taking action a at history h for a given intentional model θ, the utility of future steps U and importantly the level -1 "default" choice making model/policy π_{Default}, that starts the hierarchy. Furthermore, let $|$A(h)$|$ be the number of possible actions at history h, $|O(\{$ h, a $\})|$ be the number of possible observations after taking action a at history h, U(o) be the utility of observation o to the agent and $|$E$|$ be the number of elements of the vector E. Additionally, $\Theta(h)$ denotes the belief state of the agent at history h, the probability distributions on the possible partner types, with θ denoting a concrete intentional model and $|\Theta|$ denoting the number of intentional models that an agent holds and $p(\theta, \Theta(h))$ denotes the probability of θ under the given Belief state $\Theta(h)$. θ' denotes the intentional model that the agent themselves is using (in this concrete case, their utility and irritability) and π_ι denotes the irritation policy, with ν_ι^θ denoting the irritation weight under a given current model.

The target of the algorithm 1 (procedure ToM-HIERARCHY) is to calculate, for a given number of future steps P, all encountered histories h and ToM-levels k, the action probabilities $\mathbb{P}$(h,P,k, θ', $\Theta(h)$) for all possible actions. This is accomplished by means of action values/conditional reward expectations Q(h, P, k,θ', $\Theta(h)$) obtained by means of dynamic programming/the Bellman equation (procedure BELLMAN). Probabilities are assumed to be obtained via a logistic softmax with the action values as input (procedure PROB). The values crucially depend on the probabilities of partner actions (at level k-1) $\mathbb{P}(\{$h , a $\}$, P, k$-1, \theta$', $\Theta(h))$ (procedure OBSERVATION). "Partner-Irritation" denotes the current irritation state of the partner (as by the mechanism in the main text) under a given intentional model.

The algorithm starts from levels ≥ 0, if they are an even number of levels apart from the target level k. The reason for this is, that an agent at level k models their partner at level $k-1$ and this partner in turn models the agent at level $k-2$.

The well known Bellman equation allows to calculate current action preferences based on potential future outcomes, which in our case crucially depend on the choice preferences of the partner Q($\{$h, a $\}$, P, k -1) and the resulting likelihood $\mathbb{P}(\{$h , a $\}$, P, k-1) of choosing a response (which is included in the observation o) to the action a of the agent. So in one Bellman calculation, both the choice preference of the agent level k and the partner model at $k-1$ are being calculated.

Essentially now the partner model at $k-1$ depends only on the choice preferences of the $k-2$ (and lower) agent models, which at that point have been fully calculated and stored. In turn the level k agent can be fully calculated from the level $k-1$ response preferences alone, leading to the stated linear complexity in k.

The principles behind this algorithm are not limited to exact calculations (which may be forbiddingly expensive in larger problems). Instead the procedure could be combined with approximate solutions methods if one utilizes a "convergence criterion" for each levels' calculations, before moving to the next "level-layer". One potential complication to be aware of in this case, is that higher level simulations may choose very different action paths, therefore necessitating a return to lower level calculations, if the resulting history was not sufficiently explored at the lower level. This may incur additional costs, compared to a pure linear increase in running time as is the case for the exact calculation.

Algorithm 1 Theory of Mind Calculation

```
procedure TOM-HIERARCHY(h , k, P, θ′, Θ(h))
    for LEVEL = 0, . . . , k do
        if mod( LEVEL−k, 2) = 0 then
            Q(h, P, LEVEL, θ‘, Θ(h)) = BELLMAN(h, P, LEVEL, θ‘, Θ(h))
            ℙ(h,P, LEVEL, θ‘, Θ(h)) = PROB(Q(h, P, LEVEL, θ′, Θ(h)))
        end if
    end for
    return ℙ(h, P,k,Θ(h))
end procedure
procedure OBSERVATION(h,a,P,k, Θ(h))
    if P < 0 then
        return 0
    end if
    for o = 1, . . . , |O({ h, a })| do
        p(o)← 0
    end for
    for θ = 0, . . . , |Θ({h, a})| do
        if k > −1 then
            ν_ι^θ ← Partner-Irritation
            Q({h, a }, P, k −1, θ, Θ({h, a})) = BELLMAN({ h, a }, P, k-1, θ, Θ({h, a}))
            ℙ({ h, a }, P,k −1, θ, Θ({h, a})) = PROB( Q({h, a }, P, k −1, θ, Θ({h, a})))
            ℙ({ h, a }, P,k −1, θ, Θ({h, a})) = (1 − ν_ι^θ) ℙ({ h, a }, P,k −1, θ, Θ({h, a})) + ν_ι^θ π_ι
            for o = 1, . . . , |O({ h, a })| do
                p(o) ← p(o) + p(θ, Θ({h, a}))ℙ({h , a }, P, k−1, θ, Θ({h, a}))(o)
            end for
        else
            for o = 1, . . . , |O({ h, a })| do
                p(o) ← p(o) + p(θ, Θ({h, a}))π_Default(o)
            end for
        end if
        return p
    end for
end procedure

procedure BELLMAN(h, P, k, θ, Θ(h))
    if P < 0 then
        for a = 1, . . . , |A(h)| do
            E(a)← 0
        end for
        return E
    end if
    for a = 1, . . . , |A(h)| do
        E(a) ← 0
        p = OBSERVATION(h, a, P, k, Θ(h))
        for o = 1, . . . , |O({ h, a })| do
            h′ ← {h,a,o}
            E(a) = E(a) + r(a,o, θ) p(o)
            U(o) = BELLMAN(h′, P−1, k, θ , Θ(h′))
        end for
        ℙ =PROB(U)
        for o = 1, . . . , |O({ h, a })| do
            E(a) = E(a) + U(o)ℙ(o)
        end for
    end for
    return E
end procedure

procedure PROB(E)
    Sum ← 0
    for a = 1, . . . , |E| do
        Sum = Sum + e^E(a)
    end for
    for a = 1, . . . , |E| do
        ℙ(a) = e^E(a) / Sum
    end for
    return ℙ
end procedure
```

The various critical scalings are shown in Fig S7A-D: Fig S7A;B show the linear growth of computation time and memory respectively with (maximal) theory of mind level (including all lower-level calculations and tree storage). Conversely, Fig S7C shows the exponential rise in time for calculating a level $k^I = 4$ investor, level $k^T = 3$ trustee interaction at different planning horizons. Similarly, the exponential growth of memory use in the planning horizon for a level $k^I = 4$ investor, level $k^T = 3$ trustee can be seen in Fig S7D.

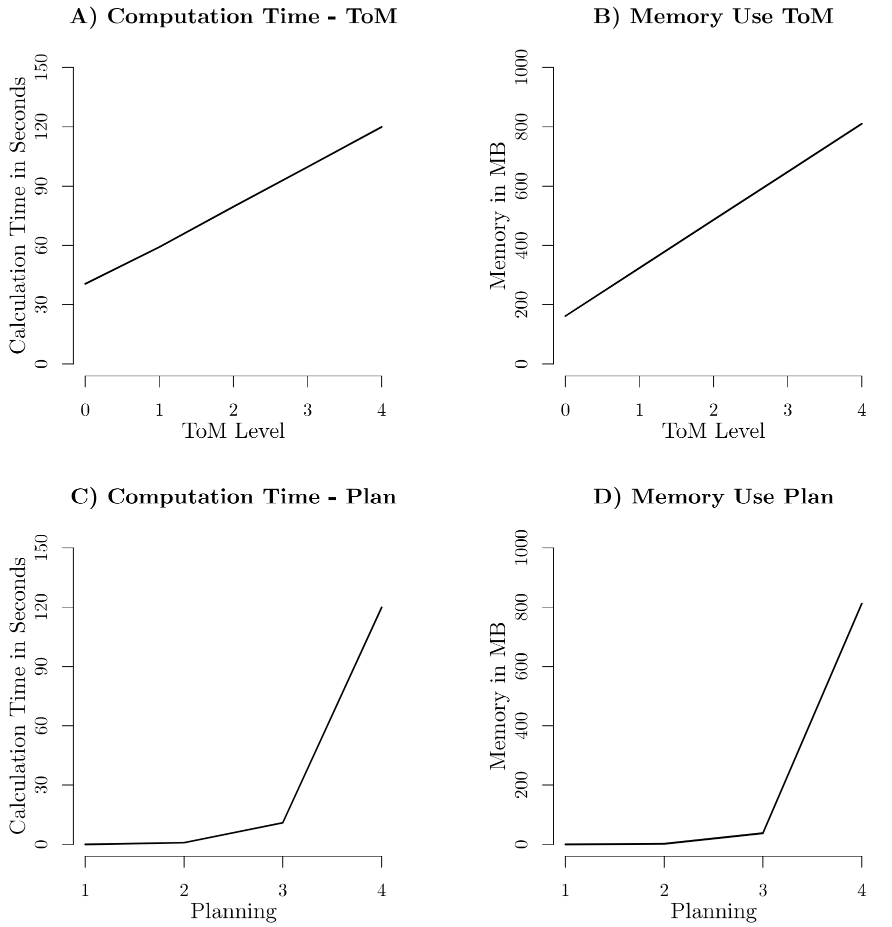

Fig. S7: A: Linear scaling of calculation time with the maximum ToM level used, at planning horizon 4. B: Linear scaling of memory use with the maximum ToM level used, at planning horizon 4. C: Exponential scaling of the computation time for a 10 step simulated interaction at ToM 4 with the planning horizon. D: Exponential scaling of the memory use for a 10 step simulated interaction at ToM 4 with the planning horizon.

Predictive Validity through Comparison with other Games

To assess the predictive validity of our estimated parameters we used another data set ($n = 178$, healthy subjects), collected in Roanoke, Virginia, at the Virginia Tech Carilion Research institute. On these subjects we have data from both the MRT, in which subjects exclusively played the investor role, and an ultimatum game, in which subjects played the role of responder.

The structure of the ultimatum game is such that one player (called "proposer") may decide to split a given amount (20 in this case) between themselves and the other player (called "responder"). The responder is presented with the proposed split and can then decide to accept, in which case

each player receives the proposed amount, or reject, in which case neither player gets anything, the proposed split.

Subjects played 60 exchanges in the role of responder in this particular paradigm. Subjects were told they would be playing a new partner every round, to avoid reputation building with a individual partner and offers and responses becoming static.

We analyzed the choice of the responder using a simple Fehr-Schmidt type utility, to express the value U of accepting an offer o (encoded as the amount proposed to the responder), given an aversion to disadvantageous inequality (called "envy") $\rho \in [0, 1]$:

$$U(o) = o - \rho \max\{10 - o, 0.0\}. \tag{10}$$

We use 10 as a reference point, since this would correspond to a fair split.

This utility is then used in a logistic softmax, with inverse temperature $\beta_u \in [0, 1]$, to determine the acceptance probability $\mathbb{P}[o]$:

$$\mathbb{P}[o] = \frac{e^{\beta_u U(o)}}{1 + e^{\beta_u U(o)}}. \tag{11}$$

Subject parameters are then determined through minimizing the negative loglikelihood of the observed acceptance and rejection time series under the generative model.

Comparing the parameters we obtained in both games through correlational analysis, we found a significant linear correlation between the risk aversion parameter ω of a subject in the MRT and both the envy ρ (Pearson correlation of 0.16, significance $p = 0.028$, uncorrected) and inverse temperature β_u (Pearson correlation of -0.158, significance $p = 0.036$, uncorrected). We found that subjects who invested large amounts in the partner in the trustgame tended to accept more (hence also lower) offers in the ultimatum game, potentially optimizing their outcome in both games. The negative correlation with inverse temperature in the ultimatum game suggests that those who accepted lower offers would stay true to this policy throughout the game.
